# The Origin and Early Radiation of Archosauriforms: Integrating the Skeletal and Footprint Record

**DOI:** 10.1371/journal.pone.0128449

**Published:** 2015-06-17

**Authors:** Massimo Bernardi, Hendrik Klein, Fabio Massimo Petti, Martín D. Ezcurra

**Affiliations:** 1 MuSe–Museo delle Scienze, Trento, Italy; 2 School of Earth Sciences, University of Bristol, Bristol, United Kingdom; 3 Saurierwelt Paläontologisches Museum, Neumarkt, Germany; 4 School of Geography, Earth and Environmental Sciences, University of Birmingham, Birmingham, United Kingdom; University of Utah, UNITED STATES

## Abstract

We present a holistic approach to the study of early archosauriform evolution by integrating body and track records. The ichnological record supports a Late Permian–Early Triassic radiation of archosauriforms not well documented by skeletal material, and new footprints from the Upper Permian of the southern Alps (Italy) provide evidence for a diversity not yet sampled by body fossils. The integrative study of body fossil and footprint data supports the hypothesis that archosauriforms had already undergone substantial taxonomic diversification by the Late Permian and that by the Early Triassic archosauromorphs attained a broad geographical distribution over most parts of Pangea. Analysis of body size, as deduced from track size, suggests that archosauriform average body size did not change significantly from the Late Permian to the Early Triassic. A survey of facies yielding both skeletal and track record indicate an ecological preference for inland fluvial (lacustrine) environments for early archosauromorphs. Finally, although more data is needed, Late Permian chirotheriid imprints suggest a shift from sprawling to erect posture in archosauriforms before the end-Permian mass extinction event. We highlight the importance of approaching palaeobiological questions by using all available sources of data, specifically through integrating the body and track fossil record.

## Introduction

Archosauriforms (crocodiles, birds, and multiple extinct taxa) became one of the most successful tetrapods on land during the Mesozoic, radiating into virtually all habitats in the aftermath of the end-Permian mass extinction event (EPME) [1]. The crown clade Archosauria comprises Pseudosuchia (all forms closer to crocodiles than to birds) and Avemetatarsalia (all forms closer to dinosaurs and birds than to crocodiles) [2–6], which together constitute one of the most taxonomically diverse clades of extant amniotes with about 10,000 species. Current continuous research efforts by both palaeontologists and molecular biologists seek for a better understanding of the early evolution of archosaurs, and specially the timing of evolutionary events and the macroevolutionary history of the group. Reliable knowledge of their early palaeobiogeography can provide hints about their plesiomorphic ecological preferences and, if compared with an independent source of data, may reveal biases in the fossil record. Finally, the understanding of the origin of archosauriforms is essential for depicting the dawn of dinosaur evolutionary history because features and fate of the first archosauriforms would have eventually set the course for the evolution of the Dinosauria [4,7].

The origin and early diversification of archosauriforms can only be comprehensively studied by looking at all available sources of palaeontological evidence. However, the possible contribution of ichnological data has rarely been taken into account when considering archosauriform origins. No recent macroevolutionary analyses on this topic use ichnology as a source of data [1,5,8,9], with the notable, but single, exception of the debate on dinosauromorph and dinosauriform origins (e.g., [10,11]). Trace fossils are considerably more abundant than body fossils, and are often preserved in those depositional environments not appropiate for the preservation of body fossils. Historical ichnological investigation has usually been based on extramorphological (substrate-related) rather than anatomical features, preventing many researchers from considering the track record as a reliable source of data.

Furthermore ichnological data often allow only a coarse taxonomic assignement and are therefore usless in fine-scale integrative analysis. Only a few recent studies have analyzed trackmacker identity based on synapomorphies identified in the skeletal record. When a cladistic approach is used, as first advised by Olsen ([12,13]; see also [14–16]), trace fossil occurrences can potentially be used as a reliable source of data in macroevolutionary studies, such as on biomechanics and locomotion (e.g., [17,18]), palaeobiogeography and palaeoecology (e.g., [19,20]), timing of evolutionary events [10], and other fundamental palaeobiological aspects, as recently reviewed by Bernardi et al. [21].

In this framework, we review Late Permian and Early Triassic archosauriform evolutionary history, considering both ichnological and body fossil records. We describe new specimens and re-interpret previously published records that allow us to document the presence of archosauriform footprints in the Late Permian of the southern Alps. These indentifications are based on a synapomorphy-based approach and represent the oldest archosauriform fossil tracks documented globally. We also discuss their implications in the light of the oldest skeletal taxa, which have a comparable age [1,22–25]. Finally, we integrate track and body fossil records for the Early Triassic with the aim of building a holistic (“total-evidence”) understanding of the early evolutionary history of Archosauriformes.

## Materials and Methods

Seven chirotheriid and chirotheriid-like manus and pes imprints from the Upper Permian of the southern Alps, northeastern Italy (PZO 5753 NMS 1235, MGR 0032, N.S. 34/82, R 6, MUSE 7446, NMS 1; PZO and NMS: Museo di Scienze Naturali dell’Alto Adige/Naturmuseum Südtirol, Bolzano/Bozen, Italy; MGR, Museo Geologico di Redagno, Bolzano/Bozen, Italy; N.S. and R: Museum of Paleontology of the "Sapienza" University of Rome, Rome, Italy; MUSE, Museo delle Scienze di Trento, Trento, Italy) were studied at first hand in their repositories. These specimens are the only Permian tracks currently known (i.e., published) worldwide that can be referred unambiguously to an archosauriform trackmaker (see discussion below). Specimen PZO 5753 is the best-preserved Permian footprint described here (1A–B) and was collected from the Deutschnofen/Nova Levante locality within the Arenaria di Val Gardena Formation (see S1 Text for other names of this formation), in the southern Alps region of the Bolzano Province (northeastern Italy). This locality is situated just a few kilometers from the well-known Bletterbach Gorge fossil locality, where all the other specimens here described were found. The footprint-bearing levels can be dated as late Capitanian to Changhsingian [26] or Lopingian (Visscher, in [27]) based on palynological evidence (see S1 Text for more details). Tracks were mapped using the conventional method of tracing footprint outlines on transparent acetate overlays. Photographs were taken under artificial light conditions for documentation. Descriptions and measurements follow standard procedures and terminology of Leonardi and Thulborn [28,29]. Some specimens labeled as 75/18, 73/93/A12 and 75/2 in Conti et al. ([30]: Figs 20, 21, 24, pls. 6(1), 8(2)) were deposited in the collections of the N.S. and/or IGPF (Museum of Paleontology and Prehistory "Piero Leonardi", University of Ferrara, Ferrara, Italy), but they could not be re-located during our study conducted between 2013 and 2014, and are probably lost. Their published photographs and drawings were used here for comparative purposes.

## Results

### The Permian Track Record

The Palaeozoic archosauriform track record is very scarce; it is restricted to a few chirotheriid footprints from the Lopingian of the Dolomites region of the southern Alps (NE Italy) that are discussed here (see also [30]). Supposed archosauriform footprints from the Late Permian of Morocco [31] have recently been redated as Early Triassic [32].

A rich diversity of tetrapod trackways associated with a well preserved macroflora has been described in the last decades from the Upper Permian Dolomites (e.g., [30,33–42]). Conti et al. [30] suggested the presence of possible “archosaur footprints” from the Arenaria di Val Gardena of the Bletterbach (see below), but these authors did not describe them in detail, nor did they deal with their evolutionary implications. The same specimens (73/93/A12, 75/2) were subsequently cited in multiple papers (e.g., [40,43–45]) but, again, never discussed in the context of archosauromorph evolution. We redescribe and discuss here these specimens and document new footprints using a synapomorphy-based approach for their attribution to trackmaker(s) [*sensu* 14].

#### Restudy of previously reported chirotheriid tracks from the Arenaria di Val Gardena

**Fig 1:**
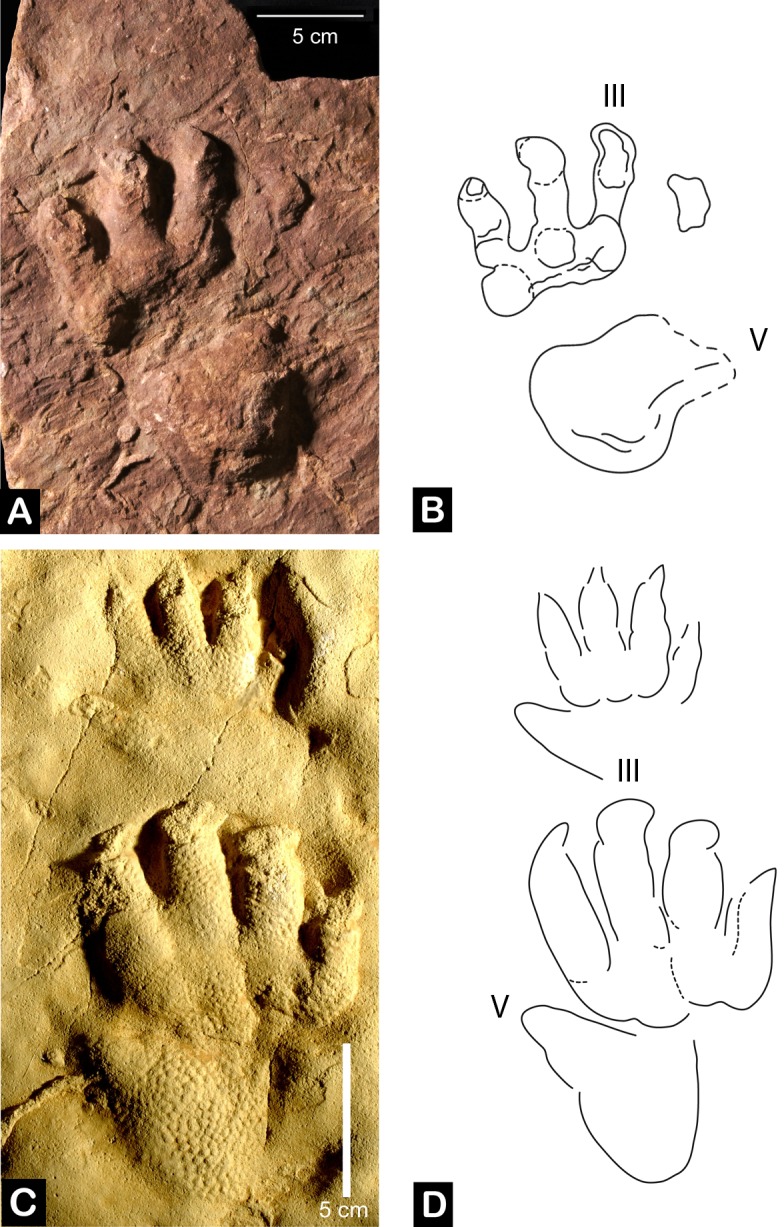
Pes and manus imprints of cf. *Protochirotherium*. A, left pes imprint PZO 1111 from the Arenaria di Val Gardena (Upper Permian) of the Deutschnofen/Nova Levante locality in northern Italy preserved as a natural cast. B, interpretative drawing. C, *Protochirotherium wolfhagense* pes-manus set (Holotype) from the Detfurth Formation (Lower Triassic, Olenekian) of Germany. D, interpretative drawing.

**Fig 2:**
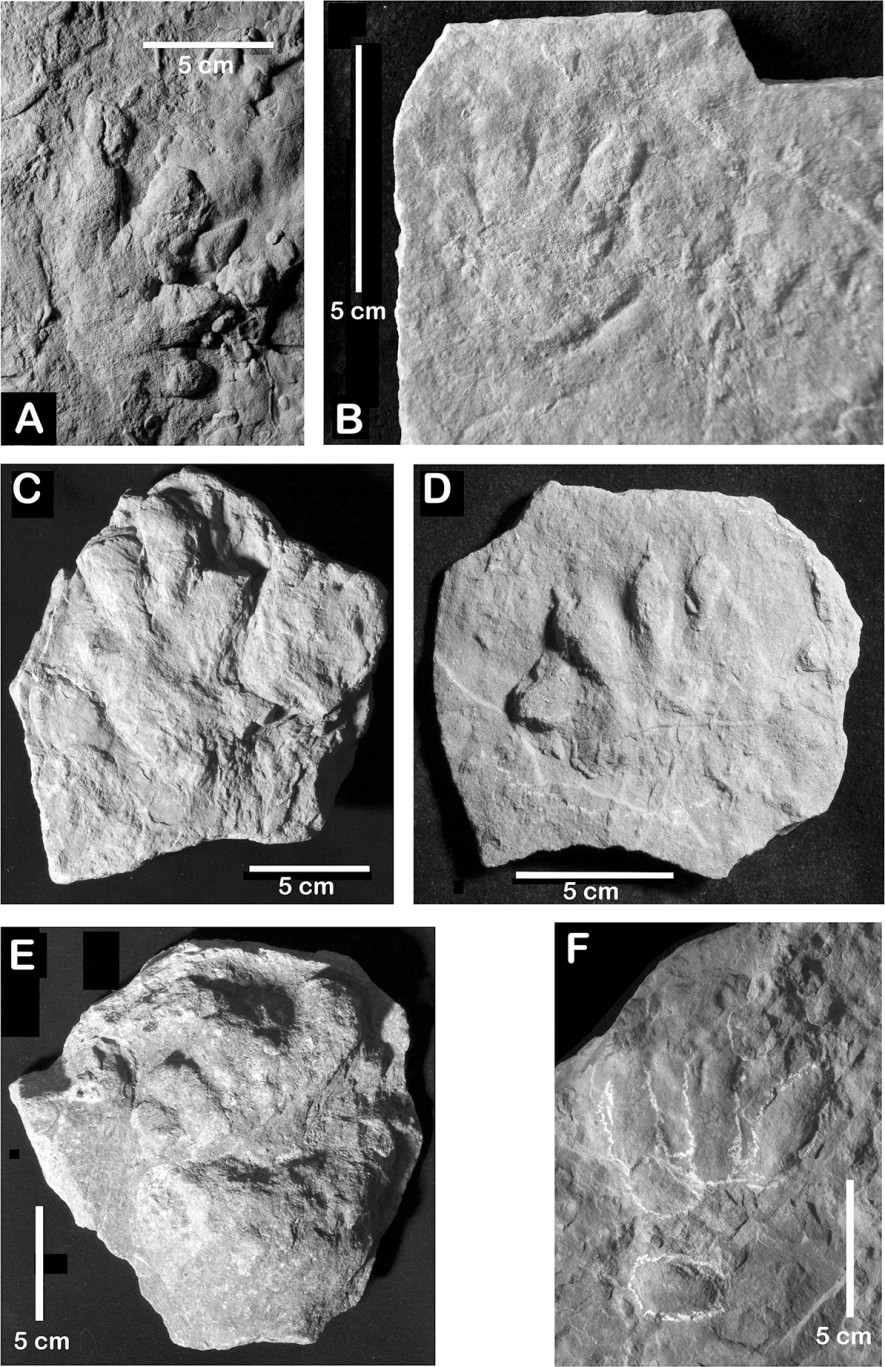
Chirotheriid and possible chirotheriid pes and manus imprints from the Arenaria di Val Gardena (Upper Permian) of northern Italy. A, possible manus imprint MGR 0032. B, pes or manus imprint N.S. 34/82. C, pes or manus imprint NMS 1235 (Inv. No. 2498). D, pes or manus imprint R6. E, deeply impressed pes imprint MUSE 7446. F, tetradactyl pes or manus imprint NMS 1.

PZO 5753 is the natural cast of a left footprint figured by Wopfner ([46]: Fig 2), but was never properly described (Fig 1A). It is 17.0 cm in length and 12.5 cm in width. The robust digits show rounded phalangeal pads and metatarsophalangeal pads proximally, forming a posteriorly concave margin. Digits increase in length from I to III. Only the distal portion of digit IV is preserved and its position indicates that it was slightly shorter than digit III. Digits I–III possess moderate divarication angles, whereas digit IV is more strongly abducted laterally. All digits terminate in rounded distal tips. Digit V consists of a massive subtriangular basal pad with a narrowing, laterally curved distal portion. Clawmarks are visible on digits I−III and they are robust and sub-triangular with a blunt tip. Digits and clawmarks possess medial and lateral striations, documenting the dynamics and movement of the pes in the substrate. All features, especially the shape of digit V and relative length of digit IV, closely resemble those observed in *Protochirotherium* [47], an ichnogenus particularly abundant in the Lower Triassic deposits of central Europe (Germany and Poland) and northern Africa [32,47–49]. Chirotheriid apomorphies are present in this specimen (e.g., massive posterolateral digit V, compact digit group I–IV), allowing an attribution to the ichnofamily Chirotheriidae [50]. However, the absence of an associated manus imprint prevents an unambiguous assignment to the ichnogenus level. PZO5753 is therefore assigne to cf. *Protochirotherium* (see S2 Text).

Conti et al. ([30]; see also [43]) assigned three footprints (75/18; 73/93/A12; 75/2) from the Bletterbach locality to “archosaurian trackmakers” ([30]: Figs 20, 21, 24, pl. 6(1), pl. 8(2)). These footprints were identified as “Proterosuchia” indet., *Synaptichnium* isp., and *Chirotherium* isp., respectively. Specimen 75/18 assigned to “Proterosuchia” indet. is not diagnosable beyond a diapsid non-chirotherian pes imprint: the arrangement of digit group I−IV with IV>III>II>I is plesiomorphic for diapsids, and a small, straight digit V is not consistent with a chirotheriid identification. The identification of *Synaptichnium* isp. for specimen 73/93/A12 cannot be evaluated here because there was no photograph published by Conti et al. [30] and the specimen could not be re-located during this study. As for specimen 75/2 ([30]: pl. 6(1)), the compact digit group I–IV and the posterolaterally oriented and recurved massive digit V, is typical for chirotheriids. The interpretative drawing of Conti et al. [30: Fig 24] differs in several aspects from the published photograph of the specimen ([30]: plate 6(1)), and only digit V seems to reflect a congruent morphology in the photograph and drawing. Digit lengths increase from I to III, with digit IV (3.2 cm) being considerably shorter than digit III (4.2 cm) and even digit II (3.84 cm). Distal ends of digits have a blunt shape, with exception of that of digit I that is distinctly tapering. Further observations cannot be made based on the published photograph. Although the absence of the manus imprint prevents any definitive assignement, this footprint closely resembles those of the ichnogenus *Protochirotherium*.

**Fig 3:**
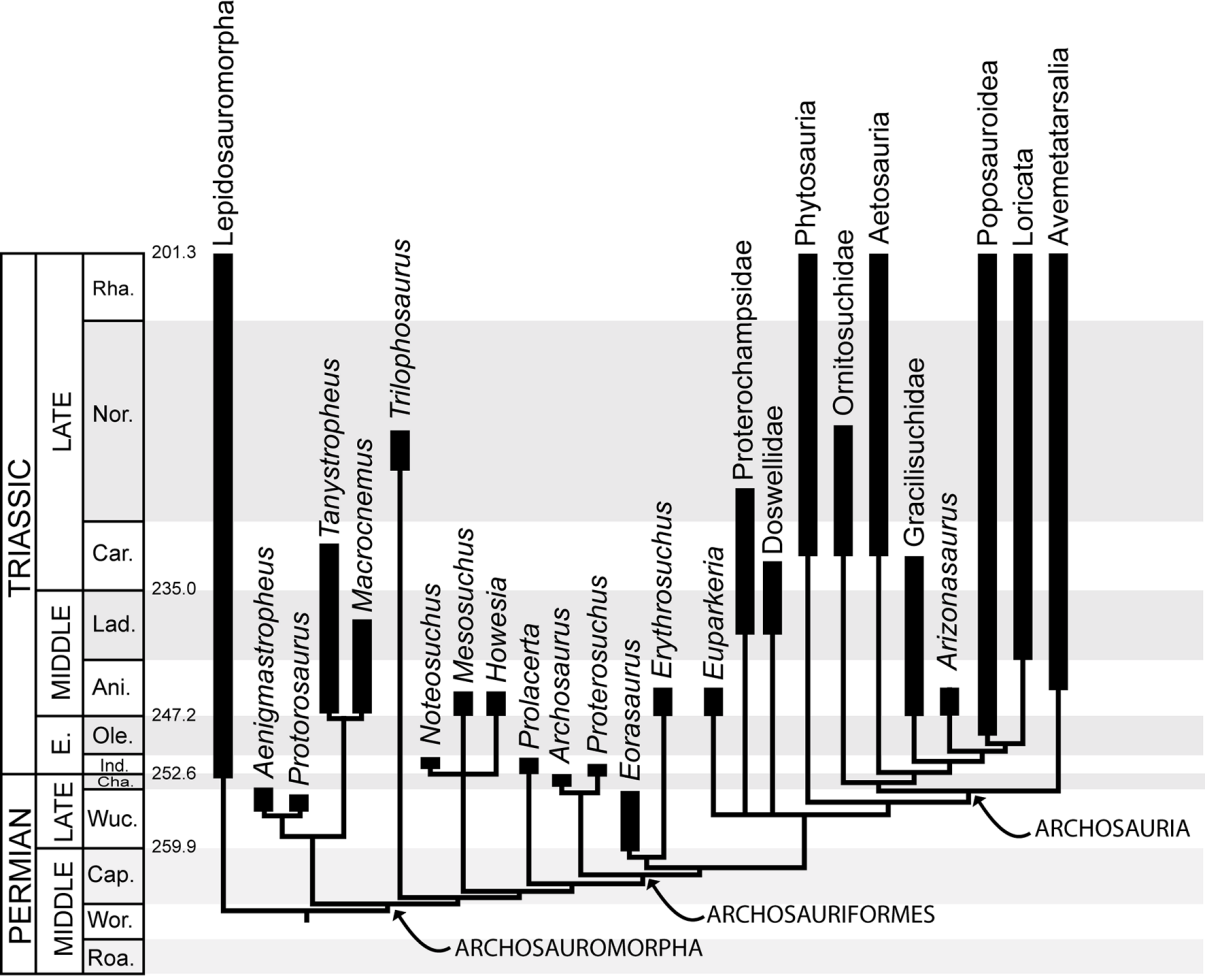
Time-calibrated cladogram of archosauromorhs discussed in the text based on skeletal remains only. Highly debated relatioships have been collapsed into polytomies. We here depict their position after [1,9,118–123]. Geological timescale after [193].

A track from the Deutschnofen/Nova Levante locality NMS 1235 (Fig 2C) was originally figured by Wopfner ([46]: Fig 3). It is a fragmentary, strongly deformed tetradactyl-pentadactyl imprint of 12 cm in length and 8.5 cm in width, and represents a manus or pes with broad digits. The middle digit (probably digit III) possesses a blunt distal end whereas the thinner? medially positioned digit? I is tapering. All the other digits are only partially preserved. Because of its incompleteness, digit proportions are unknown. No definitive chirotheriid apomorphies can be recognized in this imprint, but the overall shape suggests a chirotheriid affinity.

#### New chirotheriid material from the Arenaria di Val Gardena Formation

There are several other imprints from the Arenaria di Val Gardena Formation of the Bletterbach canyon that show chirotheriid morphology. They represent pes and manus imprints, but their isolated preservation prevents an unequivocal ichnotaxonomic attribution.

1. An isolated tetradactyl imprint (MGR 0032) (Fig 2A) is fan-shaped, short and broad. It possesses broad, robust digits finishing in blunt distal ends, with the exception of digit II that tapers distally. Digits increase in length from I to IV and digit IV is the longest. The proximal margin of the imprint is posteriorly convex. The proportionally short imprint resembles those of chirotheriid manus imprints, although no definitive assignement can be made.
2. N.S. 34/82: a pentadactyl, semi-plantigrade to plantigrade imprint with a length of ca. 6 cm (Fig 2B). Digits are relatively broad and rounded, with narrow claw traces. Digit I is the shortest and digit III the longest of the imprint. Digit IV is slightly shorter than digit III and digit V has a posterolateral orientation. N.S. 34/82 is a manus or pes impression and its overall-shape and digit proportions suggest chirotheriid affinities, resembling *Protochirotherium*. However, no more definitive assignment can be made.
3. R6: a tetradactyl manus or pes imprint with a length of ca. 10 cm (Fig 2D). It possesses short, rounded and broad digits, narrow claw marks, and a semicircular palmar or plantar surface. This imprint is very similar to chirotheriid footprints, but the absence of measurable characters prevents an unambiguous attribution.
4. MUSE 7446: a pentadactyl, semi-plantigrade, deeply impressed footprint (Fig 2E), with a length of ca. 13cm. It possesses short and blunt digits, in which digits III and IV are the longest and subequal in length. Digit IV is laterally spread and digit V is large and laterally oriented, with a massive heel. This imprint strongly resembles a *Protochirotherium* pes because of digit proportions and the robustness of digit V.
5. NMS 1: a tetradactyl manus or pes imprint (Fig 2F) with a length of ca. 13 cm. This imprint possesses long anterior digits, probably representing digits II–IV. Digit V is posterolaterally oriented, short and oval. This specimen is a probable chirotheriid, but the absence of several characters (e.g., digit I) prevents attribution to a specific ichnogenus.

#### Trackmaker identification

It is commonly assumed that tracks can only be attributed with difficulty to their producers at a low taxonomic level [14,51]. However, there is wide consensus on the assignment of some footprint morphogroups to broad taxonomic categories (e.g., [14,52–55]) based on the physical association of track and body fossil material (e.g., [56]) or detailed comparisons between tracks and limb skeletal remains (e.g., [16,57–61]. A synapomorphy-based approach was established in the last two decades and represents a clear step forward from the previous rough shape comparisons [10,12,15,18,62–68]. This approach has considerably constrained potential trackmakers and allows testing of the taxonomic hypotheses of trackmakers.

The ichnofamily Chirotheriidae Abel, 1935 [50] comprises the ichnogenera *Chirotherium* Kaup, 1835 [69], *Brachychirotherium* Beurlen, 1950 [70], *Isochirotherium* Haubold, 1971 [58], *Synaptichnium* Nopcsa, 1923 [71], *Parasynaptichnium* Mietto, 1987 [72] and *Protochirotherium* Fichter and Kunz, 2004 [48]. These taxa have been consistently interpreted as produced by archosauriforms, including stem-archosaurs and pseudosuchians [57–59,61,73–83]. Previous studies have followed the approach of Haubold [57,58,74,75], who proposed the following characters as distinct archosaur (*sensu lato*, = archosauriform) features: (1) narrow trackway, (2) small manus relative to the pes, and (3) pes and manus imprints with compact anterior digit group I–IV and posterolaterally positioned, strongly reduced digit V. However, only character 3 represents a possible archosauromorph apomorphy.

A compact digit group I–IV is also present in non-archosauromorph diapsids, such as *Youngina* (SAM-PK-K7710, Iziko South African Museum, Cape Town, South Africa: [84]) and lepidosaurs (e.g., *Dalinghosaurus* [85]). A posterolaterally positioned and divergent metatarsal and digit V is present in lepidosaurs and archosauromorphs (e.g., *Dalinghosaurus* [85]; *Protorosaurus* [86]), and probably some enigmatic basal diapsids (e.g., kuehneosaurids [87]). Nevertheless, the combination of a posterolaterally positioned, laterally oriented and robust digit V, and a massive metatarso-phalangeal region shorter than or as long as digit I seem to be unique to archosauromorphs among diapsids. Therefore, they are useful apomorphies for the identification of archosauromorph trackmakers.

Another apomorphy useful for the identification of archosauromorph imprints is a metatarsal IV shorter than or as long as metatarsal III. This character state has been recovered as a synapomorphy of the archosauriform clade that includes *Erythrosuchus* and more crownward archosauriforms ([5]: p. 191), and metatarsal length seems to be directly correlated with digit length ([5]: p. 177).

As a result, the chirotheriid imprints from the Upper Permian of the southern Alps and other chirotheriids (i.e., *Protochirotherium*, *Brachychirotherium*, *Chirotherium*, and *Isochirotherium*, see [18]) can be confidently identified as produced by an archosauriform, based on the presence of a digit IV shorter than or as long as digit III and a proportionally short digit V. Footprints PZO 1111 and 75/2 (the latter from Conti et al. 1977) and possibly, although not conclusively, the other specimens discussed in the previous section (NMS 1235, MGR 0032, N.S. 34/82, R 6, MUSE 7446, NMS 1), represent the oldest ichnologic record of archosauriforms worldwide.

### The Early Triassic Track Record

No Induan archosauriform tracks have been reported so far, contrasting with the relatively abundant body fossil record of the group in the *Lystrosaurus* Assemblage Zone of South Africa and its penecontemporaneous strata in China. Olenekian tracks are known from central Europe (Germany, Poland), northern Africa (Morocco) and North America (western USA). Track-bearing units in Germany are the Volpriehausen, Detfurth, Hardegsen and Solling formations (middle Buntsandstein). From the Volpriehausen Formation of southern Thuringia (Germany), Gümbel [88] figured chirotheriid footprints that have been assigned to the ichnogenus *Protochirotherium* by Klein et al. [47] and Klein and Niedźwiedzki [49]. The Detfurth and Hardegsen formations yielded assemblages with archosauriform footprints, including *Protochirotherium* (the type material, and the speciemens previously referred to *Palaeochirotherium* which has been shown to be a junior synonym [49]), *Synaptichnium*, *Rotodactylus* and *Prorotodactylus* [48,89–92]. The Solling Formation, which represents the Olenekian-Anisian transition, yields a diverse archosauriform ichnofauna that has been referred to *Chirotherium barthii*, *Chirotherium sickleri*, *Brachychirotherium*, *Synaptichnium*, *Isochirotherium* and *Rotodactylus* [57,58,74,76,78]. In particular, the “Thüringischer Chirotheriensandstein” [57,58,74], a unit that represents the uppermost part of the Solling Formation, preserves most of these footprints. It has to be noted here that the identification of *Brachychirotherium* in the Early-Middle Triassic is doubtful, because of problems with the diagnosis of this taxon reported by Karl and Haubold [93] and Klein and Haubold [94].

Archosauriform tracks are also known from the upper Olenekian Wióry Formation, which crops out in the Holy Cross Mountains of Poland [95–97]. They have been revised recently by Klein and Niedźwiedzki [49], and assigned to the ichnogenera *Protochirotherium* and *Synaptichnium*. *Prorotodactylus* specimens, from the same unit and locality, have been interpreted as dinosauromorph tracks by Brusatte et al. [10] and Niedźwiedzki et al. [11] (but see [49,98,99] for different views on trackmaker attribution). Klein et al. [32] reported from the Lower Triassic Timezgadiouine Formation of Morocco tracks of *Protochirotherium* and *Synaptichnium*. From the Lower Triassic Alpine Buntsandstein of Austria, Krainer et al. [100] figured chirotheriid imprints that they assigned to aff. *Protochirotherium* and cf. *Synaptichnium*. In North America, Early Triassic archosauriform tracks are known from the Wupatki Member of the lower Moenkopi Formation of Arizona, which is most probably late Olenekian in age [101,102], although no high-resolution dating is available. These footprints have been assigned to *Chirotherium sickleri*, *Synaptichnium diabloense*, *Isochirotherium coltoni* and *Chirotherium rex* [101,102]. The Moenkopi Formation of Utah (member 6 of [103]; Lower-?Middle Triassic); preserves footprints that have been assigned to chirotheriids and are very similar to *Protochirotherium* and *Synaptichnium* [103]. In South America, Melchor and de Valais [104] reported *Brachychirotherium* isp. from the Early-Middle Triassic Tarjados Formation of Argentina, which was subsequently re-assigned to *Synaptichnium* by Klein and Lucas [105].

### The Permian the Skeletal Record

Archosauromorpha includes crown diapsids more closely related to archosaurs than to lepidosaurs [106] (Fig 3). The minimum divergence time of the group based on the body fossil record is estimated at 255.7−259.9 Ma (middle-late Wuchiapingian) ([1] see also [107]), suggesting a minimum Permian evolutionary history for the group of 3.1–8.3 Ma. Only four archosauromorph nominal species are known from Permian units: *Eorasaurus olsoni* from the late Capitanian−Wuchiapingian of Russia [1,108]; *Protorosaurus speneri* from the middle Wuchiapingian of Germany and England [109–112]; *Aenigmastropheus parringtoni* from the middle−late Wuchiapingian of Tanzania [1]; and *Archosaurus rossicus* from the Changhsingian of Russia [1,22–24,113,114] (Fig 3). *Protorosaurus* and *Aenigmastropheus* have been recovered as very basal archosauromorphs in recent phylogenetic analyses [1,112]. *Archosaurus* represents the only unambiguous Permian archosauriform to date, and its holotype (PIN 1100/55, Paleontological Institute of the Russian Academy of Sciences, Moscow, Russia: premaxilla) and several of the formerly referred specimens (e.g., PIN 1100/48: skull roof; PIN 1100/66, 66a, 66b: cervical vertebrae; PIN 1100/78: dentary) possess a morphology extremely similar to that of Early Triassic proterosuchids [1,2,22–24]. *Eorasaurus* might represent the oldest archosauriform, but the very fragmentary condition of its hypodigm and similarities with tanystropheids force us to consider this assignment as tentative [1]. Potential Permian archosauromorph cranial bones and vertebrae have been reported from Uruguay ([115]; MDE pers. obs.), but substantial debate exists about the Permian or Triassic age of the fossil-bearing unit and therefore this record should be considered temporally ambiguous [1]. Regardless, multiple phylogenetic analyses suggest that the ghost lineages of some archosauromorph groups (e.g., tanystropheids, rhynchosaurs, potentially choristoderans) should extend back into the Permian [5,116], indicating an evolutionary history of several independent lineages before the EPME that is not currently sampled in the fossil record.

**Fig 4:**
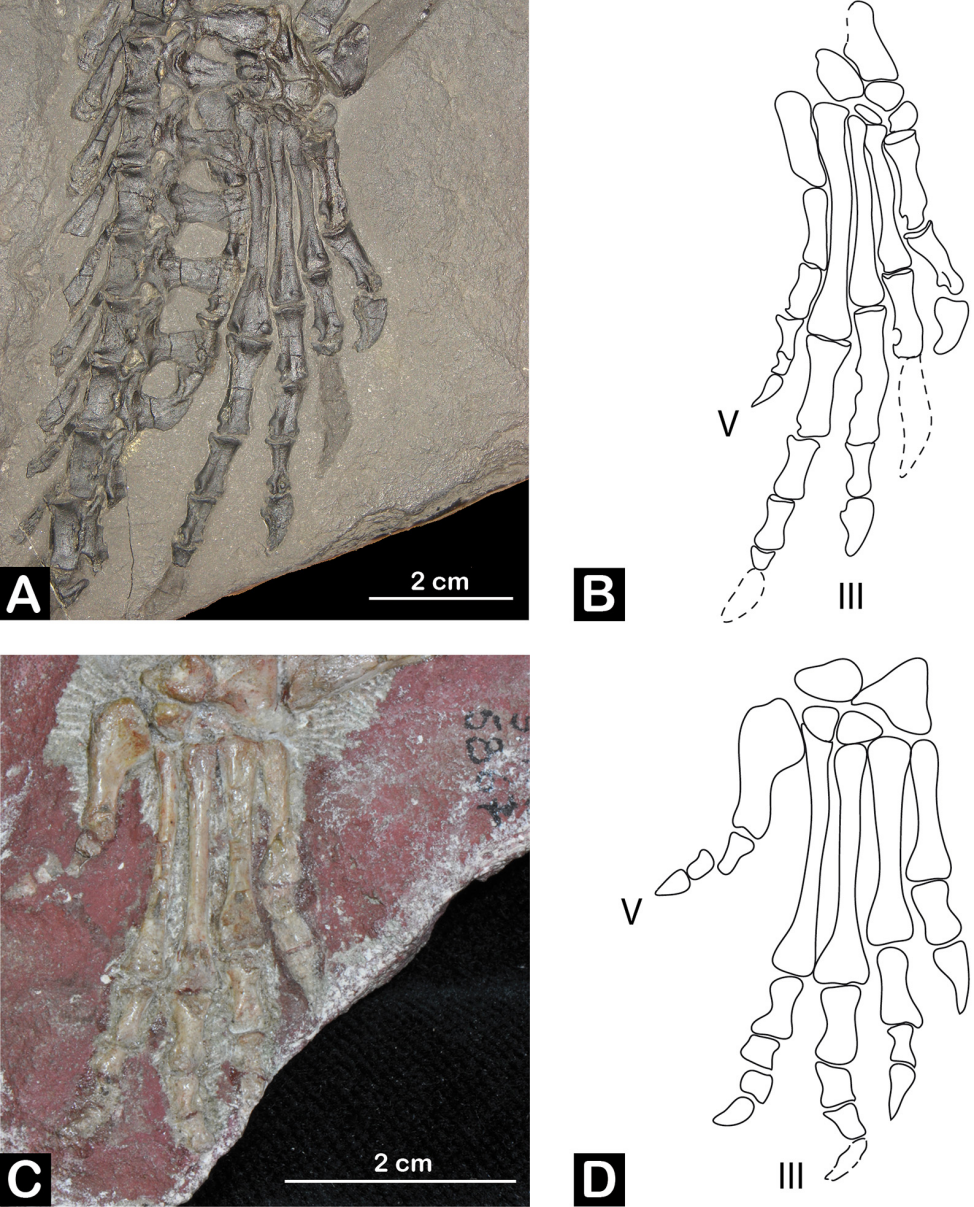
Archosauromorph foot anatomy. A, right pes skeleton of *Protorosaurus speneri* NHMW 1943I4, Naturhistorisches Museum Wien, Vienna, Austria. B, interpretative drawing. C, right pes skeleton of *Euparkeria capensis* SAM PK K8309. D, Intepretative drawing. Note that *Protorosaurus* has an ectaxonic pes, with digit IV>III>II>I while *Euparkeria* shows a mesaxonic foot with III being the longest.

Most of these Permian archosauromorphs are known from fragmentary remains, mostly restricted to the axial skeleton [1]. However, *Protorosaurus* is an exception because is known from multiple articulated specimens, including all regions of the skeleton [112]. As a result, *Protorosaurus* is particularly relevant for the integration of skeletal and ichnological data because it is the only Permian archosauromorph with known autopodia (Fig 4).

### The Early Triassic Skeletal Record

The archosauromorph record in the aftermath of the EPME is considerably more abundant and geographically widespread than that of the Permian [1,24]. Recent studies based on osteological evidence concluded that the early evolutionary radiation of archosauriforms and archosaurs occurred by the late Early Triassic or early Middle Triassic [5,117]. Nevertheless, the earliest phase of archosauriform history is still patchily understood, largely because of the paucity of the early record of several lineages [5,117,118]. In this regard, the presence of poposauroid archosaurs in uppermost Olenekian strata implies long ghost lineages, suggesting that all main archosauriform (and many archsaurian) lineages should already have been present by that time (e.g., proterochampsids, doswelliids, euparkeriids, phytosaurs, ornithosuchids, gracilisuchids, aetosaurs, and avemetatarsalians) [1,5,117–123].

Early Triassic non-archosauriform archosauromorphs are relatively taxonomically diverse and restricted to a handful of groups, including rhynchosaurs (*Noteosuchus*), ‘prolacertiforms/protorosaurs’ (e.g., *Prolacerta*, *Kadimakara*, *Boreopricea*, *Prolacertoides*, *Czatkowiella*, *Malutinisuchus*, *Vritramimosaurus*, *Augustaburiania*) and probable trilophosaurids (e.g., *Coelodontognathus*, *Vitalia*). Non-archosauriform archosauromorphs remains have been found in Lower Triassic units worldwide, namely South America, Africa, Antarctica, Europe, Asia, and Australia [1,22,25,116,124–139].

The Early Triassic archosauriform record is dominated in terms of taxonomic diversity by species historically referred to Proterosuchidae [25]. The proterosuchid record is particularly abundant in well sampled Induan−lower Olenekian beds of South Africa (*Proterosuchus fergusi*, *Proterosuchus alexanderi*, *Proterosuchus goweri* [25,140–142]). In addition, some well-preserved, articulated partial proterosuchid skeletons have been collected in approximately coeval beds of China (‘*Chasmatosaurus*’ *yuani* [126,143–145]). More fragmentary potential proterosuchids are known from the Early Triassic of Australia (*Tasmaniosaurus*, *Kalisuchus*, Proterosuchidae indet. [146–150]), Russia (*Blomosuchus*, *Vonhuenia*, *Chasmatosuchus* [24,151,152]), and India (*Ankistrodon*, ‘*Chasmatosaurus*’ sp. [153,154]).

Erythrosuchids appear for the first time in the fossil record in the Olenekian beds of Russia and South Africa (*Garjainia prima*, *Garjainia madiba* [24,25,155–157]). In addition, several Early Triassic non-archosaur archosauriforms of uncertain or problematic phylogenetic affinities have been described from South America [138,139], South Africa [158], Antarctica [159], Europe (*Osmolskina*, *Collilongus* [137,160]), and the Early−Middle Triassic of China (*Fugusuchus*, *Guchengosuchus* [161,162]). The Early Triassic archosaur record is extremely scarce when it is compared with that of non-crown archosauromorphs, being restricted to poposauroids found in the uppermost Olenekian beds of Germany and Russia (*Ctenosauriscus*, *Vytshegdosuchus* [23,24,117]), and upper Olenekian−lower Anisian beds of China (*Xilousuchus* [119]).

## Discussion

### Can Any Known Permian Archosauromorph Be the Producer of Chirotheriid Tracks?

Establishing if known Permian archosauromorphs can be considered potential candidates for *Protochirotherium*-like or chirotheriid trackmakers means establishing whether the track record supports recent fragmentary findings or provides hints to a diversity still not documented by skeletal remains. Three of the four currently recognized Permian archosauromorph species (i.e., *Archosaurus rossicus*, *Eorasaurus olsoni* and *Aenigmastropheus parringtoni*) are only known from fragmentary remains lacking autopodial bones [1,22,24,108]. The only Permian archosauromorph species with known autopodia is *Protorosaurus speneri*, a quadrupedal reptile with a body length of up to 1.5–2.0 m [112]. The foot of *Protorosaurus* possesses five metatarsals, of which metatarsal IV is the longest, followed by metatarsals III, II, I, and V, respectively. Pedal digit IV is the longest, clearly longer than III, and the pedal phalangeal formula is 2-3-4-5-4 [112]. The limb morphology of the latest Permian proterosuchid archosauriform *Archosaurus* and putative referred specimens is unknown. Nevertheless, their morphology is extremely similar to that of the earliest Triassic proterosuchids (e.g., *Proterosuchus fergusi*) and autopodial morphology may have been possibly similar to that of stratigraphically younger proterosuchids. Metatarsal IV (63.5 mm) is considerably longer than metatarsal III (57.0 mm) in *Proterosuchus fergusi* (SAM-PK-K140), resembling the condition in non-archosauriform archosauromorphs (the complete length of digit IV is unknown in collected proterosuchid specimens). In contrast, metatarsal III is as long as, or longer than, metatarsal IV in *Erythrosuchus* and more crownward archosauriforms [2,3,5,163].

Conti et al. [30] excluded *Proterosuchus* as the producer of some of the Permian tracks described here because in this taxon metatarsals I−IV strongly increase in length, suggesting footprints with a sharp cross-axis angle (i.e., defined as the angle between the metapodial-phalangeal axis and the long axis of the footprint along digit III; of the four angles formed by these two axes, the cross-axis angle is the lateral and anterior one; [28]). As a result, potential footprints of *Proterosuchus* would be strongly asymmetric or ectaxonic with digit IV being considerably longer than digit III, and with a very long digit V. In this respect they would probably be similar to those expected for *Protorosaurus* [86] (Fig 4A and 4B) and other non-archosauriform archosauromorphs. In contrast, the imprint pattern of *Protochirotherium* is mesaxonic.

The producer-taxon of *Synaptichnium* footprints had a digit IV longer than, or subequal to, digit III, resulting in a distinct ectaxonic shape of the pes imprint. Thus, early archosauromorphs, such as *Protorosaurus* [86] and *Proterosuchus* [25] are potential candidate trackmakers of *Synaptichnium* footprints, although extremely narrow *Synaptichnium* trackways, known for example from the Moenkopi Group (Lower-Middle Triassic) of Arizona ([101], [102]: p. 30, Fig 28], might contradict this possibility. Both protorosaurs/tanystropheids and proterosuchids are expected to have had more spread gaits [25,164]. Note also that digit IV subequal to digit III may indicate a trackmaker more derived than proterosuchids [25].

Klein and Niedźwiedzki [49] and Klein et al. [47] suggested erythrosuchids and/or pseudosuchian archosaurs as probable producers of Early Triassic *Protochirotherium* footprints. Limb position in early archosauriforms is poorly known, therefore any definitive conclusion cannot be reached. Nevertheless, articulated proterosuchid skeletons show a sprawling posture (e.g., ‘*Chasmatosaurus*’ *yuani*: IVPP V4067, Institute of Vertebrate Paleontology and Paleoanthropology, Beijing, China) and the very similar morphology of the acetabulum and proximal end of femur in proterosuchids and erythrosuchids suggests a similar sprawling posture in life. All known *Protochirotherium* trackways are narrow, indicating that proterosuchids and erythrosuchids might be excluded as potential producers of chirotheriid footprints because they should have left broader trackways. Accordingly, we suggest that more crownward archosauriforms are the probable trackmakers of Late Permian and Early Triassic chirotheriid and chirotheriid-like imprints. The presence of a digit IV shorter than or subequal to digit III in *Protochirotherium* indicates that the producer is an archosauriform, or even a member of Archosauria. Accordingly, the chirotheriid *Protochirotherium*-like footprints described here from the southern Alps are the oldest evidence of a mesaxonic foot within Archosauromorpha. The stratigraphically oldest archosauriform with a mesaxonic foot is *Euparkeria* from the early Middle Triassic of South Africa (see [122] and references therein; Fig 4C and 4D), but several Early Triassic archosauriforms may also have had mesaxonic feet based on their phylogenetic position (e.g., poposauroid archosaurs [5]). Interpretations of *Osmolskina czatkowicensis* as an euparkeriid [138] might imply the presence of mesaxonic taxa in the Early Triassic, but recent analyses recovered it further outside Archosauria with respect to *Euparkeria* ([120]; see also [122]).

A number of other early archosauromorph clades which have their ghost lineages extending into the considered time span, should also be considered. Among these are tanystropheids, rhynchosaurs, trilophosaurids, and proterochampsids that all have well preserved limbs allowing full comparison with the track record. Tanystropheids have digit IV > III (e.g., [165]) and would have therefore produced ectaxonic imprints. Furthermore they were mainly marine and their footprints, named *Gwyneddichnium*, are known from the Upper Triassic of Pennsylvania and Colorado [53,166]. Rhynchosaurs can also be excluded as possible trackmakers of mesaxonic tracks, as their pes have digit IV > III (e.g., [167]). Furthermore the ichnospecies *Synaptichnium pseudosuchoides* was recently assigned to rhynchosaurs by Tresise and King [168]. *Trilophosaurus*, also had an ectaxonic pes. *T*. *buettneri* [169], for example, shows a pes digit IV clearly longer than III. Lockley et al. [170] dubitatively attributed *Apatopus* ichnogenus to trilophosaurs, but more recently Padian et al. [60] discarded this possibility. When autopodia are preserved, as in *Chanaresuchus*, proterochampsids show digit III≥IV [171], an arrangement therefore compatible with a mesaxonic print. However their digit IV is extremely thin, and digit V is highly reduced, in the shape of a ‘hook’ without phalanges [3,171,172] making proterochampsids not suitable candidates as *Protochirotherium* trackmakers. Furthermore, other chirotheriid tracks, like *Isochirotherium delicatum*, have been attributed to proterochampsid trackmakers [173].

**Fig 5:**
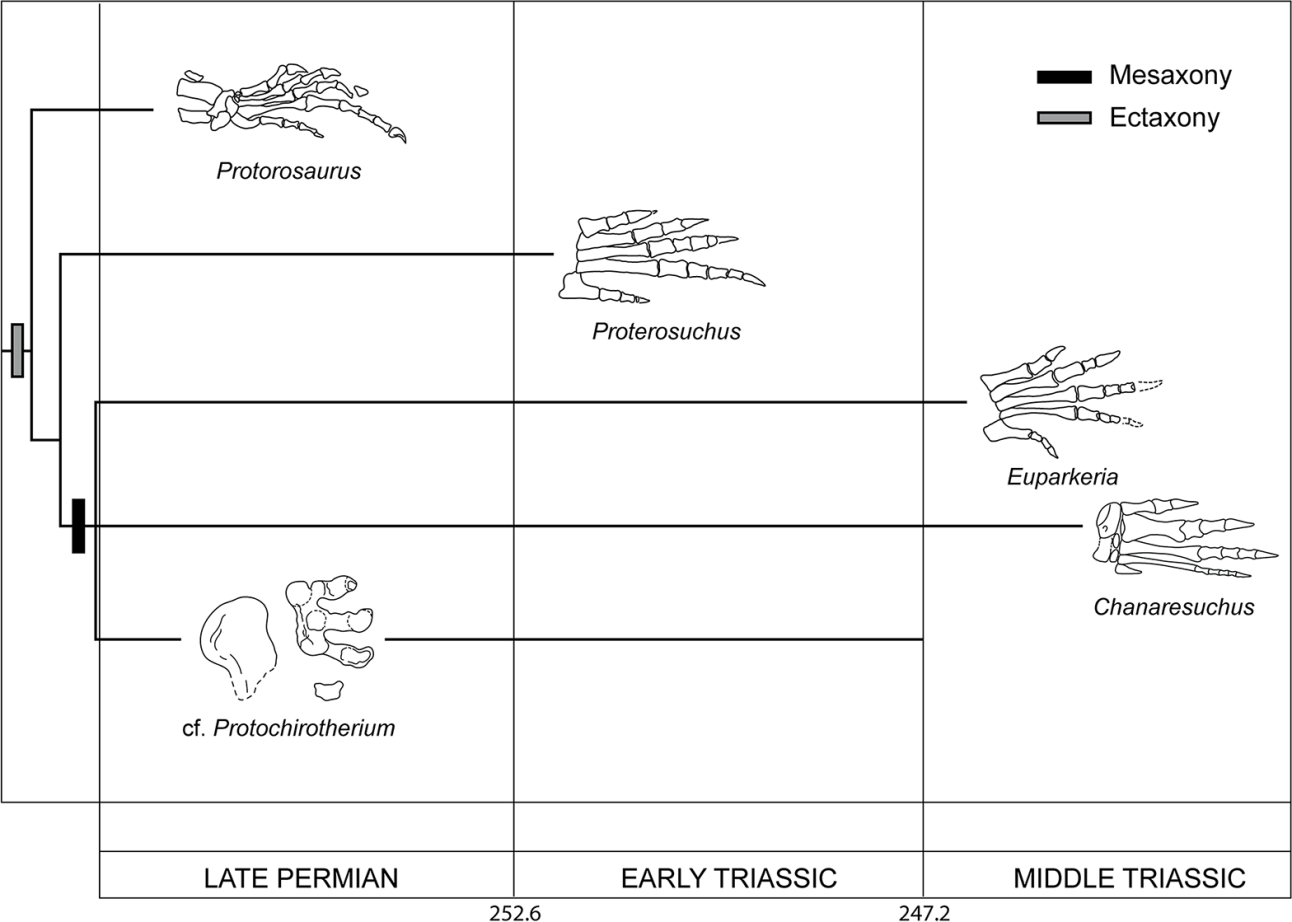
Evolution of the mesaxonic pes in archosauromorphs. Grey and black boxes indicate evolution of apomorphic characters: grey = ectaxony, black = mesaxony. Late Permian *Protochirotherium* pulls the evolution of mesaxony down the archosauriform tree and anticipates the oldest skeletal remain (*Euparkeria capensis*) by 10 Ma.

The discovery of Late Permian *Protochirotherium*–like footprints therefore represent also the oldest evidence of mesaxony, indicating the presence of archosauriforms more crownward than proterosuchids before the Permian-Triassic boundary, and predating the appearance of a mesaxonic foot in the body fossil record by ca. 10 My (Fig 5). Long ghost ranges in early archosauriform evolution were inferred because of the presence of the proterosuchid *Archosaurus* and the potential archosauriform *Eorasaurus* in the Late Permian of Russia [1]. *Eorasaurus* was recovered as more closely related to erythrosuchids and *Euparkeria* than to proterosuchids. As a result, *Protochirotherium*-like tracks provide independent evidence for a potentially taxonomically broader evolutionary radiation of archosauriforms in the Late Permian than previously recorded.

### Early Archosauriform Palaeobiogeography

**Fig 6:**
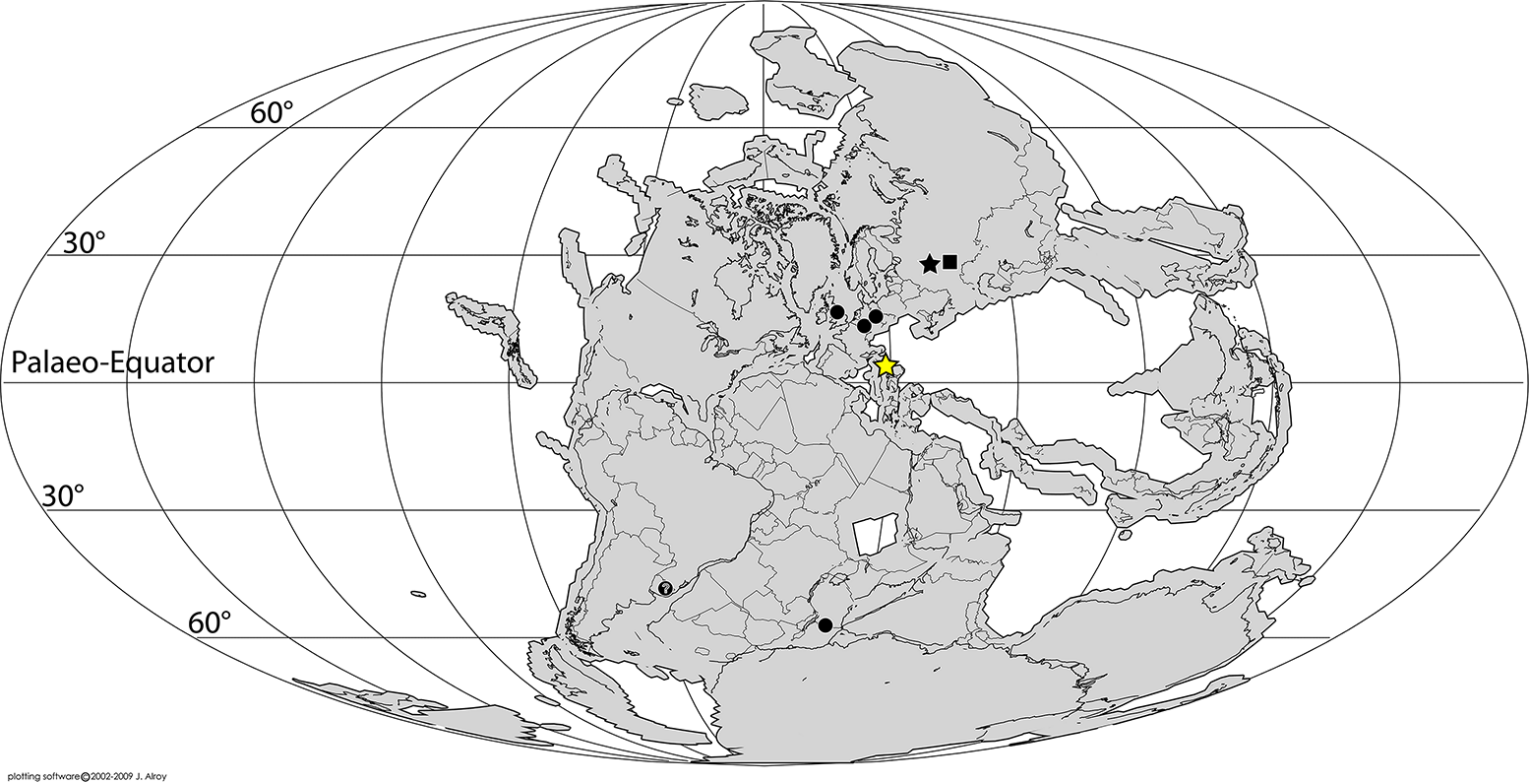
Paleogeographic distribution of Late Permian archosauriform footprints (yellow stars) and body fossil (black shapes) localities across Pangea. Squares = indeterminate archosauromorphs, circles = non-archosauriform archosauromorphs, stars = archosauriforms. Chirotheriid footprints from the southern Alps (NE Italy) document the lowest palaeolatitudinal record of archosauriforms and bridge the tropical gap in the disjunct distribution of the skeletal record. Paleomap for 260 Ma downloaded from Fossilworks using data from the Paleobiology Database [194].

A recent revision of the early archosauromorph body fossil record found a rather palaeolatitudinally broad geographical distribution for the group during the Late Permian [1]. Current evidence indicates that early archosauromorphs spanned from few degrees north to the palaeo-Equator (Germany and England) to a palaeolatitude of 30°N (Russian localities) in the northern hemisphere to high palaeolatitudes of 55°S (Tanzania) in southern Pangaea (Fig 6).

The report here of Late Permian palaeo-Equatorial archosauriforms partially bridges the gap between the northern records of archosauromorphs and that of the Southern Hemisphere, representing the palaeolatitudinally lowest occurrence of the group. In addition, these imprints extend the geographic range of archosauriforms between palaeolatitudes of 0°−30°N before the EPME.

**Fig 7:**
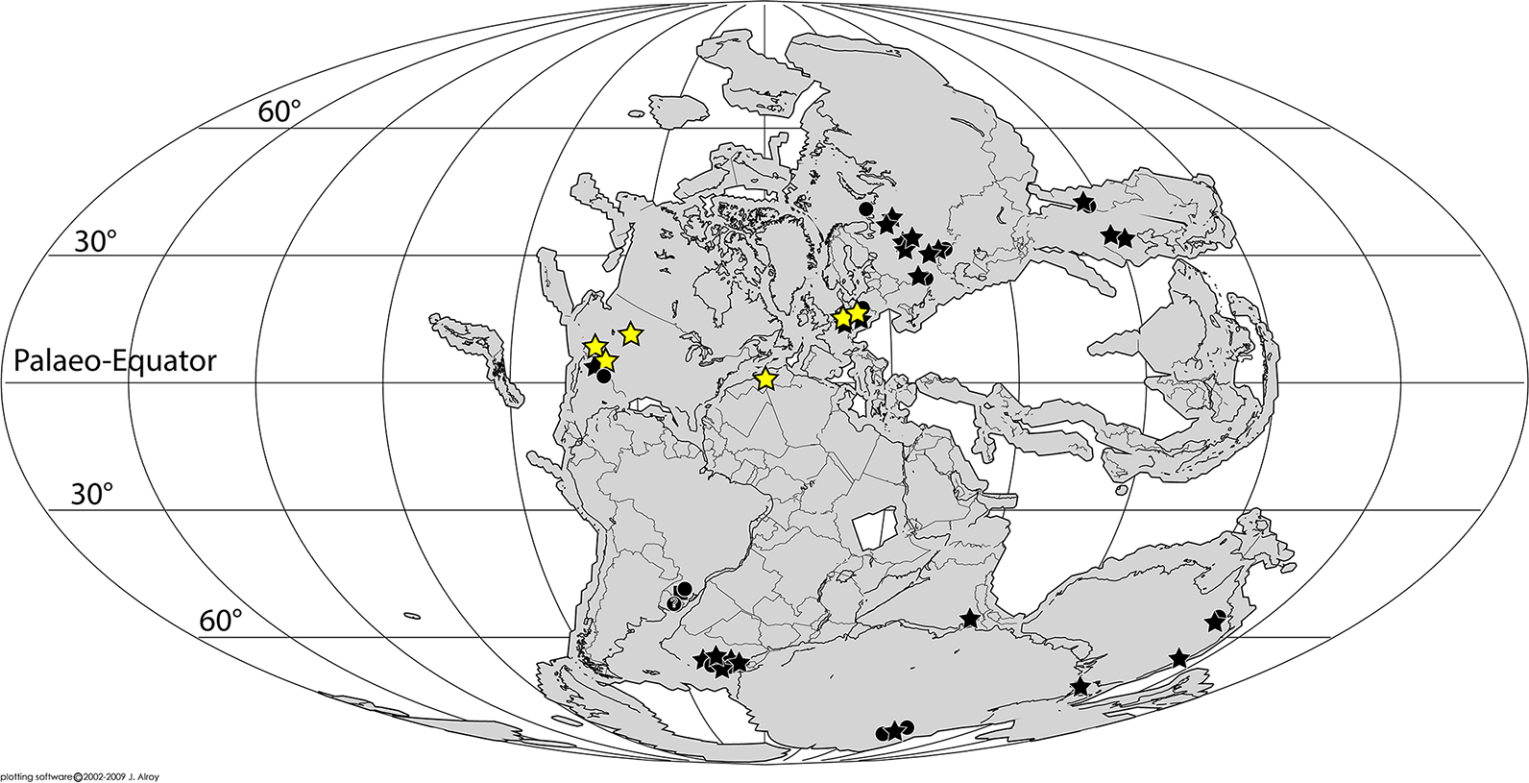
Paleogeographic distribution of Early Triassic archosauriform footprints (yellow stars) and body fossil (black shapes) localities across Pangea. Squares = indeterminate archosauromorphs, circles = non-archosauriform archosauromorphs, stars = archosauriforms. Footprints indicate that archosauriforms, soon after their origin, were distributed also at low latitudes. Paleomap for 250 Ma downloaded from Fossilworks using data from the Paleobiology Database [194].

The Early Triassic body and footprint fossil record indicates a global geographic distribution of archosauromorphs, being considerably broader than that present in the Late Permian [5,32,48,49,96,117,125,137,146,160,174–177] (Fig 7). In particular, tracksites in Arizona and Utah support the presence of archosauromorphs in central-western Pangaea during the Early Triassic, documented in the body fossil record by the single report of Nesbitt [178], while the best known evidence of the presence of the group in this region is Anisian in age (i.e., *Arizonasaurus* [179,180]).

Multiple non-archosaur archosauriform and archosaur lineages are inferred to have been present during the Early Triassic based on current phylogenetic hypotheses, and yet these remain unsampled (e.g., gracilisuchids, ornithosuchids, phytosaurs, proterochampsids [5,117,118,181]). The striking absence of crown archosaurs in the Early Triassic, with the exception of poposauroids, has led to the hypothesis that early archosaurs originated or, at least began to diversify, in areas that have a bias towards the non-preservation of body fossil remains or are not well sampled, such as the tropics [117,119]. The geographic palaeodistribution of archosauriform skeletal remains during the Early Triassic shows that most of them occur outside the tropical belt, with the sole exception of the three European taxa: *Ctenosauriscus* [117], *Osmolskina*, and *Collilongus* [137,160,166]. In contrast, archosauriform ichnosites are mainly concentrated in the tropics, namely between the palaeo-Equator and the Tropic of Cancer, in central and western Pangaea. The only possible exception is a single *Synaptichnium* footprint from northwestern Argentina [104,105], but the age of this record is poorly constrained and could be either Early or Middle Triassic. An uneven sampling or taphonomic bias are likely explanations for this distribution pattern, and future discoveries may reveal the presence of more chirotheriids at high palaeolatitudes in the northern and southern hemispheres as well as body fossils at low latitudes. Beyond these biases, footprints indicate that archosauriforms were distributed also at low latitudes soon after their origin. It is worth noting that Sun et al. [182] suggested that the absence of vertebrates from the Equatorial belt in the Early Triassic reflects lethally hot temperatures for five million years after the EPME. This conclusion however was based solely on the distribution of body fossils, and archosauriform track data reject this hypothesis.

### Body Size in Early Archosauriforms

Studies of evolutionary changes in body size have long attracted the attention of researchers. After comparing ichnological and skeletal fossil evidence, we test here the effect of the new data on previously published studies. Although Permian chirotheriid footprints are rare, the body size of the trackmaker can be compared with that of the oldest archosauriforms. We estimated an approximate body length for *Archosaurus* using a linear regression (premaxillary body height vs. skull length) composed of ten Early Triassic proterosuchid specimens (see S3 Text). The total skull length of *Archosaurus* is estimated as ca. 460 mm and closely resembles that of the largest sampled specimens of the earliest Triassic *Proterosuchus fergusi* (GHG 231, Geological Survey, Pretoria, South Africa: 477.0 mm [120]). As a result, *Archosaurus* should have reached a total body length similar to that of *Proterosuchus fergusi*, being of approximately 3–3.5 m [142]. Therefore, *Archosaurus* is considerably larger than the only known complete Permian archosauromorph, *Protorosaurus speneri*, which reached a body length of up to 1.5–2 m [112]. The total body length of the Permian chirotheriid trackmakers from the southern Alps is calculated in approximately 2 m, following estimations conducted by Gand et al. [183] for Triassic chirotheriid footprints. As a result, the body size estimated for these Permian trackmackers fits the range expected for the oldest known archosauriforms based on the body fossil record.

#### Evolutionary trends in early archosauriform evolution

Trends in archosaur body size through time have been recently investigated using both skeletal [184,185,186] and ichnological data [18]. Footprint size (commonly measured as pes length) is a reliable parameter for such analysis because it correlates directly with body size [17,29], and can be easily measured from footprints, even if they are not arranged in trackways. In addition, when considering true tracks (or shallow undertracks–about 1 cm deep, [187]), pes length is less dependent on substrate consistency and taphonomic distortion with respect to other descriptive measurements (e.g., interdigital angle; [188,189]). Using a database of 125 published trackway occurences, Kubo and Kubo [18] found a statistically significant increase in foot length between Early and Middle Triassic non-dinosauromorph archosauriforms, and interpreted the result in the context of locomotory and biomechanichal novelties that occurred during the early evolution of archosaurs.

We use here a modified version of the database of Kubo and Kubo [18] of Early and Middle Triassic chirotheriids, which was increased and updated with the addition of 17 new records from the Late Permian and Early Triassic (see S1 Table). Late Triassic occurrences were present in the original database, but they were excluded here because they are outside the main aims of our study, which are: (i) explore evolutionary trends with the addition of the Permian record and test for significative changes in size across the Permo-Triassic boundary, and (ii) test the effect of the inclusion of the so-called “Wióry Formation megaichnofauna” described by Niedźwiedzki and Ptaszyński [97] and not included in the original dataset. The megaichnofauna of the Wióry Formation represents the oldest known ichnological record of very large (ca. 6 m long) archosauriforms [97].

**Fig 8:**
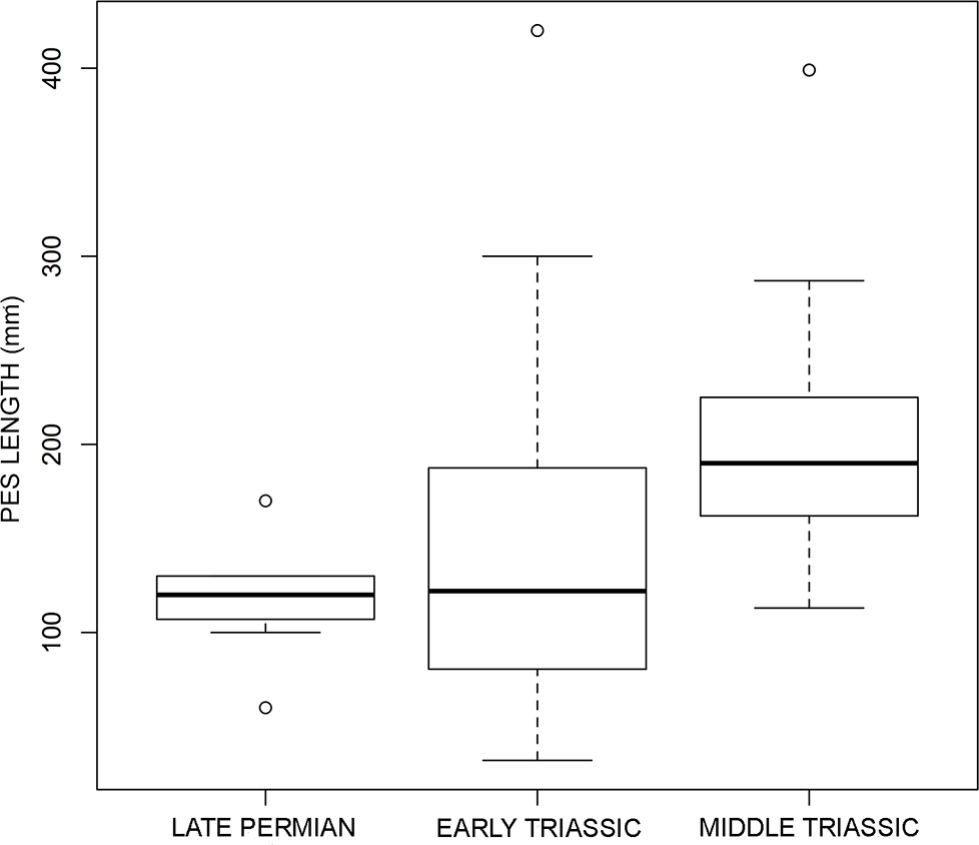
Archosauriform body size through time as derived from track length. Average track size indicates that archosauriform body size did not change significantly from the Late Permian to Early Triassic, although maximum values show a significant increase. The Permo-Triassic mass exctintion might not have affected archosauriform body size. Based on data of S1 Table.

The small sample size of the Permian archosauriform track record prevents a robust statistical analysis of the data and full comparison with that from the Early and Middle Triassic. However, until further discoveries are made, a first set of analyses is valuable when testing for patterns and trends. Permian chirotheriids (mean pes length = 117.7 mm, median = 120 mm, 3σ = 33.4) are fully within the variance of the Early Triassic sample (mean pes length = 134.4 mm, median = 122 mm, 3σ = 78.1), and the latter distribution only partially overlaps that of the Middle Triassic. A Mann-Whitney U test showed that the difference in foot length between Late Permian and Early Triassic occurences is non-significant (W = 197.5, p = 0.8594), but it is significant between the Early Triassic and Middle Triassic (W = 345, p = 0.0001), in which Middle Triassic footprints are significantly larger. Accordingly, the impact of the “Wióry megaichnofauna”on the analysis was not significant and bolsters the results recovered by Kubo and Kubo [18]. The extreme values (minumum and maximum) between Late Permian and Early Triassic ichnological samples are strikingly different, in which both upper and lower body size boundaries increased in Early Triassic archosauriforms (Fig 8). However, because the mean and median of the Late Permian sample are not significantly different from that of the Early Triassic, we can suggest that the average body size of archosauriforms did not change substantially across the Permo-Triassic boundary. However, more data is necessary for a strong test of the hypothesis that the EPME did not have a significant effect on archosauriform average body size. On the other hand, if maximum values only are taken into account, the large size of the Wióry ichnofauna [97] implies that maximum body size doubled in less than 10 My during the aftermath of the EPME.

### Palaeoecological Inferences

**Fig 9:**
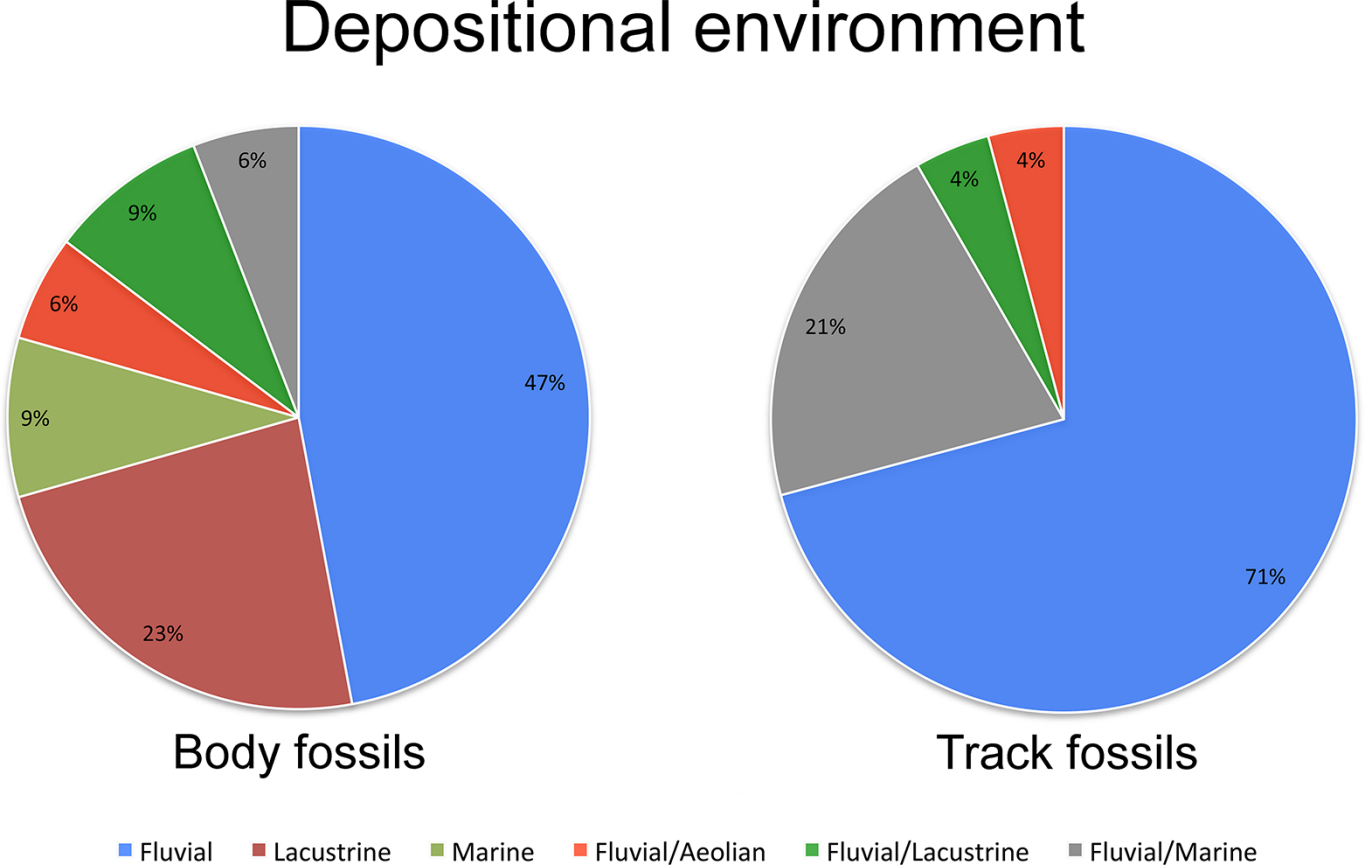
Depositional environment of late Permian and Early Triassic archosauromorph-bearing formations. The common pattern exhibited by the body fossil and the track fossil record suggest a real environmental/ecological preference for inland-fluvial (lacustrine) environments for early archosauromorphs.

Trace fossils may be preserved in environments that are not appropriate for the preservation of bones and teeth. Therefore, ichnological data can provide an independent source of information about the ecology of trackmakers, and allow evaluation of a potential bias in skeletal data (e.g., allochthonous association). To investigate this issue, the depositional environment of each Late Permian and Early Triassic geological formation that yielded skeletal and/or ichnological archosauromorph records was surveyed. Strikingly, almost all specimens, including both body and trace fossils, were found in non-marine formations, ranging from fluvial (e.g., channel and braided systems) to lacustrine environments. The only two exceptions are *Protorosaurus*, [112] and the tanistropheid *Augustaburiania* [136]. However, the occurrences of *Protorosaurus* are very likely allochthonous, because at least one specimen possesses gut contents that are terrestrial in origin [190]. Although the fossil bearing horizon is not always well constrained, resulting in uncertainties regarding the depositional environment for several taxa and ichnotaxa (e.g., fluvial/lacustrine, fluvial/aeolian) both body fossils and tracks fossils were mostly found in fluvial sediments (respectively 47% and 71% in our database, that when all possible fluvial influence on the depositional environment is considered goes up to 68% and 100%, respectively; see S2 Table for more details; Fig 9). Interestingly, no track fossil is known from lacustrine deposits (or max 4% if the fluvial/lacustrine uncertainties are considered) while 23% of body fossils come from this depositional environment. We interpret this datum as a preservational bias. The whole predominance of early archosauromorphs in fluvial and lacustrine rocks may in fact reflect a preservational bias, as suggested for other taxa (see [191] and references therein). However, the common pattern exhibited in both the skeletal and track record may suggest a real environmental/ecological preference for inland-fluvial (lacustrine) environments for early archosauromorphs. The occurrence of early archosauromorphs in multiple fluvial (to lacustrine) subenvironments (e.g., floodplains, braided river systems) with a broad palaeolatitudinal range implies that they lived inland, inhabiting freshwater continental environments, irrespective of the local palaeoclimate, and they possibly possessed broad climatic tolerance.

### Locomotory Features

The acquisition of an upright limb posture and a parasagittal gait was a key innovation in early archosauriform evolution [17,57,58,60,74,75]. The position of the legs under the body enabled fast running with low loss of energy compared with the gait of typical sprawlers. The classical view, expressed by Charig [192], mantained that Early Traissic archosaurs (*sansu lato*) were sprawlers, Middle Triassic ones were semi-erect (or “semi improved”, as he termed it), and fully erect gait emerged in the late Middle to Late Triassic. However, in an analysis of compiled fossil trackway data, Kubo and Benton [16] showed that archosauriforms with erect posture originated and became common in the Early Triassic. The parasagittal gait is reflected in the narrow trackways of all chirotheriids, with occasional long strides [57,58,75]. Chirotheriid trackways are all characterized by a narrow gauge [18,57,58] and all known Early Triassic *Protochirotherium* trackways show a similar condition (see [49]: Figs 9, 12, 15). No unambiguous chirotheriid trackways have so far been discovered in the Late Permian of the southern Alps, but it is more parsimonious to hypothesise that Permian chirotheriid trackways would show the same pattern exhibited by all Triassic chirotheriids. *Protochirotherium*–like tracks from the southern Alps can therefore be considered as the earliest indirect evidence for narrow-gauge trackmakers, which walked with an erect gait. This suggests that at least some archosauriforms may have adopted an erect gait during the Late Permian. Together with the trends observed in some therapsid lineages [17], Late Permian *Protochirotherium*-like tracks may therefore support an earlier shift from a sprawling to an erect posture in archosauriforms, raising questions about the conclusions of Kubo and Benton [17], who linked the shift to the Permo-Triassic event. However, this conclusion does not contradict the hypothesis that erect walkers radiated soon after the mass extinction [17], and discoveries of Palaeozoic chirotheriid trackways are strictly necessary to support any conclusion on this issue.

## Conclusions

The integrative study of body and track records allows a better understanding of the origin of archosauriforms. The ichnological record supports a Late Permian–Early Triassic radiation of archosauriforms not well documented by skeletal material, but implied by ghost ranges deduced from the most recent phylogentic analyses and supported by the recent recovery of the Late Permian *Eorasaurus* as a possible non-proterosuchid archosauriform. Newly studied footprints from the southern Alps provide evidence of a Late Permian diversity not yet sampled by body fossils, which widens the geographical distribution of this clade before the Permo-Triassic boundary. Studied tracks provide evidence of several morphologically distinct archosauriform groups in central Pangaea in the Late Permian–Early Triassic and suggests that this region might be crucial also for future discoveries of body fossil remains.

The integration of footprint and body fossil data sheds light on early archosauriform evolution and suggests that:

1. Archosauriforms had already undergone substantial taxonomic diversification by the Late Permian. *Eorasaurus* (a derived archosauromorph with no appendicular skeleton preserved) and cf. *Protochirotherium* tracks are independent evidence of a taxonomically broader evolutionary radiation of archosauriforms in the Late Permian than currently expected.
2. The integration of body and track data suggests a broader geographical distribution of Early Triassic archosauromorphs. Footprints support body fossil data by indicating that archosauriforms were distributed also at low latitudes soon after their origin.
3. Tracks indicate that the archosauriform body size did not change significantly from the Late Permian to Early Triassic. The possibility that the Permo-Triassic event did not affect substantially archosauriform body size constitutes a new hypothesis that should be tested in the future with more data both from the body and footprint record.
4. Skeletal and track record suggest an environmental/ecological preference for inland fluvial (lacustrine) environments for early archosauromorphs. The broad palaeolatitudinal range occupied implies broad climatic tolerance.
5. Late Permian *Protochirotherium*-like imprints might support a shift from a sprawling to an erect posture in archosauriforms before the Permo-Triassic event (contra Kubo and Benton [16]). Althoug no Palaeozoic chirotheriid trackways are known to date, this constitute a new working hypothesis that will be tested as new specimens become available.

## Supporting Information

S1 Table Late Permian to Middle Triassic archosauriform track length database used in the statistical analysis. (DOCX)

S2 Table Depositional environment of the fossil-bearing formations. (XLSX)

S1 Text Geology and age of the Arenaria di Val Gardena Formation. (DOCX)

S2 Text Ichnostratigraphic remarks. (DOCX)

S3 Text *Archosaurus rossicus* body length estimation. (DOCX)
