# Supplementary material for: The Origin and Early Radiation of Archosauriforms: Integrating the Skeletal and Footprint Record
Source: PLoS One. 2015 Jun 17;10(6):e0128449. doi: 10.1371/journal.pone.0128449 (PMC4471049; doi:10.1371/journal.pone.0128449)
Supplement: S1 Table — (DOCX) [file pone.0128449.s001.docx]

**Supporting information**

Bernardi et al. The origin and early radiation of archosauriforms: integrating the skeletal and footprint record. PlosOne.

**S4**

Late Permian to Middle Triassic archosauriform track length database used in the statistical analysis

Modified from Kubo and Kubo (2013). Lines in bold indicate newly added data. See Materials and Methods section for more details. TL: Track Length. Note that, following Kubo and Kubo (2013), we consider the speciemens described by Haubold (1967, 1971a,b) as Early Triassic although the exact age of the “Thüringischer Chirotheriensandstein” of Haubold is uncertain. Considering that the uppermost part of the Solling Formation is Early Anisian (see Bachmann and Kozur, 2004) the age of the specimens could be either late Early or early Middle Triassic.

| **Reference paper** | **Ichnotaxon** | **TL** | **Age** |
| --- | --- | --- | --- |
| Avanzini and Leonardi (2002) | *Isochirotherium inferni* | 205 | Middle Triassic |
| Courel and Demathieu (1976) | *Brachychirotherium circaparvum* | 113 | Middle Triassic |
| Courel and Demathieu (1976) | *Brachychirotherium circaparvum* | 173 | Middle Triassic |
| Courel and Demathieu (1976) | *Isochirotherium felenci* | 205 | Middle Triassic |
| Courel and Demathieu (1976) | *Isochirotherium felenci* | 247 | Middle Triassic |
| Courel and Demathieu (1976) | *Isochirotherium felenci* | 286 | Middle Triassic |
| Courel and Demathieu (1976) | *Isochirotherium felenci* | 287 | Middle Triassic |
| Demathieu (1974) | *Chirotherium gallicum* | 175 | Middle Triassic |
| Demathieu (1974) | *Chirotherium barthii* | 198 | Middle Triassic |
| Demathieu (1984) | *Brachychirotherium gallicum* | 160 | Middle Triassic |
| Demathieu (1984) | *Brachychirotherium gallicum* | 162 | Middle Triassic |
| Demathieu (1984) | *Brachychirotherium gallicum* | 174 | Middle Triassic |
| Demathieu (1984) | *Brachychirotherium gallicum* | 179 | Middle Triassic |
| Demathieu and Durand (1991) | *Chirotherium mediterraneum* | 190 | Middle Triassic |
| Haderer et al. (1995) | *Chirotherium sickleri* | 145 | Middle Triassic |
| Haderer et al. (1995) | *Chirotherium sickleri* | 150 | Middle Triassic |
| Haderer et al. (1995) | *Isochirotherium felenci* | 190 | Middle Triassic |
| Haderer et al. (1995) | *Isochirotherium felenci* | 217 | Middle Triassic |
| Haubold (1971) | *Chirotherium lomasi* | 180 | Middle Triassic |
| Peabody (1948) | *Isochirotherium marshalli* | 225 | Middle Triassic |
| Peabody (1948) | *Chirotherium barthii* | 135 | Middle Triassic |
| Peabody (1948) | *Chirotherium barthii* | 225 | Middle Triassic |
| Lopez (1993) | *Brachychirotherium cf. gallicum* | 399 | Middle Triassic |
| Demathieu and Fichter (1989) | *Chirotherium sickleri* | 81 | Early Triassic |
| Demathieu and Haubold (1982) | *Brachychirotherium kuhni* | 98 | Early Triassic |
| **Fichter and Kunz (2004)** | ***Protochirotherium wolfhagense*** | **109** | **Early Triassic** |
| Fuglewicz et al. (1990) | *Isochirotherium sanctacrucense* | 113 | Early Triassic |
| Haubold (1967) | *Brachychirotherium praeparvum* | 32 | Early Triassic |
| Haubold (1967) | *Brachychirotherium harrasense* | 35 | Early Triassic |
| Haubold (1967) | *Brachychirotherium harrasense* | 38 | Early Triassic |
| Haubold (1967) | *Chirotherium soergeli* | 40 | Early Triassic |
| Haubold (1967) | *Brachychirotherium harrasense* | 40 | Early Triassic |
| Haubold (1967) | *Isochirotherium soergeli* | 44 | Early Triassic |
| Haubold (1967) | *Isochirotherium soergeli* | 47 | Early Triassic |
| Haubold (1967) | *Chirotherium sickleri* | 47 | Early Triassic |
| Haubold (1967) | *Brachychirotherium praeparvum* | 48 | Early Triassic |
| Haubold (1967) | *Brachychirotherium praeparvum* | 49 | Early Triassic |
| Haubold (1967) | *Chirotherium sickleri* | 70 | Early Triassic |
| Haubold (1967) | *Chirotherium sickleri* | 80 | Early Triassic |
| Haubold (1971a) | *Chirotherium hildburghausense* | 70 | Early Triassic |
| Haubold (1971a) | *Chirotherium sickleri* | 78 | Early Triassic |
| Haubold (1971a) | *Chirotherium sickleri* | 83 | Early Triassic |
| Haubold (1971a) | *Chirotherium sickleri* | 85 | Early Triassic |
| Haubold (1971a) | *Chirotherium sickleri* | 92 | Early Triassic |
| Haubold (1971a) | *Chirotherium sickleri* | 100 | Early Triassic |
| Haubold (1971a) | *Chirotherium sickleri* | 123 | Early Triassic |
| Haubold (1971a) | *Chirotherium sickleri* | 130 | Early Triassic |
| Haubold (1971a) | *Isochirotherium soergeli* | 140 | Early Triassic |
| Haubold (1971a) | *Isochirotherium soergeli* | 143 | Early Triassic |
| Haubold (1971a) | *Chirotherium sickleri* | 155 | Early Triassic |
| Haubold (1971a) | *Chirotherium jenense* | 170 | Early Triassic |
| Haubold (1971a) | *Chirotherium hessbergense* | 180 | Early Triassic |
| Haubold (1971a) | *Chirotherium bipedale* | 180 | Early Triassic |
| Haubold (1971a) | *Chirotherium barthii* | 185 | Early Triassic |
| Haubold (1971a) | *Chirotherium barthii* | 190 | Early Triassic |
| Haubold (1971a) | *Chirotherium barthii* | 195 | Early Triassic |
| Haubold (1971a) | *Chirotherium barthii* | 195 | Early Triassic |
| Haubold (1971a) | *Chirotherium barthii* | 195 | Early Triassic |
| Haubold (1971a) | *Chirotherium barthii* | 195 | Early Triassic |
| Haubold (1971a) | *Chirotherium barthii* | 200 | Early Triassic |
| Haubold (1971a) | *Chirotherium barthii* | 200 | Early Triassic |
| Haubold (1971a) | *Chirotherium barthii* | 210 | Early Triassic |
| Haubold (1971a) | *Chirotherium barthii* | 220 | Early Triassic |
| Haubold (1971a) | *Chirotherium barthii* | 225 | Early Triassic |
| Haubold (1971a) | *Chirotherium barthii* | 235 | Early Triassic |
| Haubold (1971a) | *Isochirotherium herculis* | 280 | Early Triassic |
| Haubold (1971a) | *Isochirotherium herculis* | 300 | Early Triassic |
| **Haubold (1971b)** | ***Synaptichnium*** | **100** | **Early Triassic** |
| **Klein et al., 2010** | ***Protochirotherium-Synaptichnium*, CDUE60** | **160** | **Early Triassic** |
| **Klein et al., 2010** | ***Protochirotherium-Synaptichnium*, CDUE61** | **100** | **Early Triassic** |
| **Klein et al., 2010** | ***Protochirotherium-Synaptichnium*, CDUE64** | **170** | **Early Triassic** |
| **Klein et al., 2010** | ***Protochirotherium-Synaptichnium*, CDUE66** | **90** | **Early Triassic** |
| **Niedźwiedzki and Ptaszyński, 2007** | ***Synaptichnium senkowiczowae*** | **300** | **Early Triassic** |
| **Niedźwiedzki and Ptaszyński, 2007** | ***Brachychirotherium kalkowensis*** | **420** | **Early Triassic** |
| Peabody (1948) | *Chirotherium sickleri* | 85 | Early Triassic |
| Peabody (1948) | *Chirotherium sickleri* | 94 | Early Triassic |
| Peabody (1948) | *Chirotherium minus* | 122 | Early Triassic |
| Peabody (1948) | *Isochirotherium coltoni* | 122 | Early Triassic |
| Ptaszyński (2000) | *Synaptichnium kotanskii* | 64 | Early Triassic |
| Ptaszyński (2000) | *Isochirotherium sanctacrucense* | 108 | Early Triassic |
| **Tourani et al., 2010** | **cf. *Synaptichnium*** | **135** | **Early Triassic** |
| **Tourani et al., 2010** | ***Synaptichnium* cf. *pseudosuchoides*** | **136** | **Early Triassic** |
| **Conti et al., 1977 as reviewed in this work** | **cf. *Protochirotherium*** | **114** | **Late Permian** |
| **This work** | **Chirotheriidae indet., NMS 1235** | **120** | **Late Permian** |
| **This work** | **Chirotheriidae indet., MUSE 7446** | **130** | **Late Permian** |
| **This work** | **Chirotheriidae indet., N.S. 34/82** | **60** | **Late Permian** |
| **This work** | **Chirotheriidae indet., NMS 1** | **130** | **Late Permian** |
| **This work** | **Chirotheriidae indet., R6** | **100** | **Late Permian** |
| **Wopfner, 1999 as reviewed in this work** | **cf. *Protochirotherium*** | **170** | **Late Permian** |

References

Avanzini M, Leonardi G (2002) *Isochirotherium inferni* ichnosp. n. in the Illyrian (Late Anisian, Middle Triassic) of Adige Valley (Bolzano, Italy). Boll Soc Pal It 41: 41–50.

Bachmann GH, Kozur HW (2004) The Germanic Triassic: correlations with the international chronostratigraphic scale, numerical ages and Milankovitch cyclicity. Hall Jb Geowiss B26: 17−62.

Conti MA, Leonardi G, Mariotti N, Nicosia U (1977) Tetrapod footprints of the “Val Gardena Sandstone” (North Italy). Their paleontological, stratigraphic and paleoenvironmental meaning. Palaeont It NS 40: 1−91.

Courel L, Demathieu G (1976) Une ichnofauna reptilienne remarquable dans les gres Triasiques de Largentiere (Ardeche, France). Palaeontographica Abt A 151: 194–216.

Demathieu GR (1974) Les dalles a empreintes de pas de reptiles du museum d'histoire naturelle de Lyon. N Arch Mus Hist Nat Lyon 12: 5–12.

Demathieu GR (1984) Une ichnofauna du Trias Moyen du Bassin de Lodeve (Herault, France). Ann Paleont (Vert.-Invert.) 70: 247–273.

Demathieu GR, Durand M (1991) Les traces pas de Tetrapodes dans le Trias detrique du Var et des Alpes-Maritimes (France). Bull Mus Nation Hist Nat C 13: 115–133.

Demathieu G, Fichter J (1989) Die Karlshafener Fährten im Naturkundemuseum der Stadt Kassel. Philippia 6: 111–154.

Demathieu G, Haubold H (1982) Reptilfährten aus dem Mittleren Buntsandstein von Hessen (BRD). Hallesches Jahrb 7: 97–109.

Fichter J, Kunz R (2004) New genus and species of chirotheroid tracks in the Defurth Formation (Middle Bunter, Lower Triassic) of Central Germany. Ichnos 11: 183–193.

Fuglewicz R, Ptaszyński T, Rdzanek K (1990) Lower Triassic footprints from the Swietokrzyskie (Holy Cross) Mountains, Poland. Acta Pal Pol 35: 109–164.

Haderer F-O, Demathieu GR, Böttcher R (1995) Wirbeltier-Fahrten aus dem Rötquarzit (Oberer Buntsandstein, Mittlere Trias) von Herdheim bei Wertheim/Main (Süddeutschland). Stuttgarter Beit Nat B 230: 1–31.

Haubold H (1967) Eine Pseudosuchier-Fährtenfauna aus dem Buntsandstein Südthürringens: Hall JB Mitteldeut Erdgesch 8: 12–48.

Haubold H (1971a) Die Tetrapodenfährten des Buntsandsteins in der Deutschen Demokratischen Republik und in Westdeutschland und ihre Äquivalente in der gesamten Trias. Paläeont Abhand Abteil A 4: 397–548.

Haubold H (1971b) Ichnia Amphibiorum et Reptiliorum fossilium. Handbuch der Paläoherpetologie, 18: 1–124.

Klein H, Voigt S, Hminna A, Saber H, Schneider J, Hmich D (2010) Early Triassic Archosaur-Dominated Footprint Assemblage from the Argana Basin (Western High Atlas, Morocco). Ichnos 17: 215–227.

Kubo T, Kubo M (2013) Analysis of Triassic archosauriform trackways: difference in stride/footprint ratio between dinosauromorphs and other archosauriforms. Palaios 28: 259–265.

Lopez AP (1993) Estudio de las huellas de reptil, del ichnogenero *Brachychirotherium*, encontradas en el Trias Subbetico de Cambil. Estud Geol 49: 77–86.

Niedźwiedzki G, Ptaszyński T (2007) Large Chirotheriidae tracks in the Early Triassic of Wiory, Holy Cross Mountains, Poland. Acta Geol Pol 57: 325–342.

Peabody FE (1948) Reptile and amphibian trackways from the Lower Triassic Moenkopi Formation of Arizona and Utah. Uni Cal Bull Dep Geol Sci 27: 295–468.

Ptaszyński T (2000) Lower Triassic vertebrate footprints from Wióry Holy Cross Mountains, Poland. Acta Pal Pol 45: 151–194.

Tourani A, Benaouiss N, Gand G, Bourquin S, Jalil N-E, Broutin J, Battail B, Germaine D, Khaldoune F, Sebban S, Steyer J-S, Vacante R (2010) Evidence of an Early Triassic age (Olenekian) in Argana Basin (High Atlas, Morocco) based on new chirotherioid traces. Compt Rend Palevol 9: 201–208

Wopfner H (1999) Über Tetrapoden-Fährten, Kohlen und versteinerte Hölzer aus dem Grödner Sandstein (Perm) bei Deutschnofen. Der Schlern 73: 23–32.
