# Supplementary material for: The Origin and Early Radiation of Archosauriforms: Integrating the Skeletal and Footprint Record
Source: PLoS One. 2015 Jun 17;10(6):e0128449. doi: 10.1371/journal.pone.0128449 (PMC4471049; doi:10.1371/journal.pone.0128449)
Supplement: S1 Text — (DOCX) [file pone.0128449.s003.docx]

**Supporting information**

Bernardi et al. The origin and early radiation of archosauriforms: integrating the skeletal and footprint record. PlosOne.

**S1**

Geology and age of the Arenaria di Val Gardena

New findings and previously described ichnological specimens discussed in this paper were found in the Upper Permian Arenaria di Val Gardena, also known in the literature as Gröden Formation or Val Gardena Sandstone of the Dolomites region, Southern Alps, NE Italy. This formation was originally described as the Grödener Sandstein by Richthofen (1860). It was studied in detail from a stratigraphic and sedimentologic point of view by Massari et al. (1988, 1994), Massari and Neri (1997) and recently revised by Neri (2007). The Arenaria di Val Gardena crops out between the Eastern part of the Southern Alps, between the Trentino Alto-Adige and Friuli Venezia Giulia regions. It is a succession of continental red beds, up to 600 meters thick, whose deposition was strongly controlled by the irregular paleotopography of the Athesian Volcanic Group. Indeed, the sedimentation of the Arenaria di Val Gardena started after a long period of subaerial erosion (Morelli et al., 2007) with a sedimentary hiatus that lasted approximately from 14 to 27 million years (Cassinis et al., 1999). The Arenaria di Val Gardena is mainly characterized by decimeter-scale to meter-scale beds of red-greyish siliciclastic sandstone interbedded with centimeter to decimeter levels of red-greyish siltstones, marly siltstones and marls. The base of the unit is characterised by poorly sorted conglomerates, deriving from the erosion of the underlying substrate (Massari et al., 1988; Neri, 2007). Marly/silty dolomite as well as sparse nodules or continuous layers of gypsum occasionally occurs within the red marly intervals. Calcic paleosol beds (caliche, calcrete) may also occur within the formation. The most common sedimentary structures are large-scale cross-lamination, parallel lamination, ripple marks and desiccation structures (mud-cracks). The lithic fragments include volcanics (ignimbrites and lavas of andesitic to rhyolitic composition), granitoids and medium grade metamorphic rocks (Neri, 2007). In the easternmost sector, the Arenaria di Val Gardena is interbedded with the marine Bellerophon Formation, mainly comprising evaporites and shallow water carbonates.

The Arenaria di Val Gardena reflects an alluvial plane environment, characterized in the distalmost sector by extensive alluvial fans, braided and meandering rivers, coastal plain and a terminal fan. Paleocurrent patterns indicate that the rivers entered from the northern and western raised sectors and were directed toward the east (Massari & Neri, 1997). Marine influence is testified by the increase of bioturbation and occurrence of flaser wavy bedding, as well as the 2 meter thick marine horizon recorded in the famous Bletterbach gorge succession (Cassinis et al. 1999; Broglio Loriga et al., 1988; Avanzini et al., 2010). These evidences suggest a tidally influenced coastal plain (Avanzini et al., 2010). The occurrence of gypsum nodules and layers as well as calcic paleosols testify to coastal sabkha and evaporitic lagoons and warm to hot, semi-arid climate (Ori, 1988; Massari et al., 1988; Cassinis et al., 1999).

The Arenaria di Val Gardena rests unconformably on the Athesian Volcanic Group or on the metamorphic basement and passes upward and laterally to the Bellerophon Formation, strongly charcaterized by evaporitic and lagoon deposits (Neri, 2007). In the westermost sector, the Arenaria di Val Gardena is overlain by marine deposits of the Werfen Formation.

The Arenaria di Val Gardena has long been known for its abundance of tetrapod footprints (Leonardi, 1948; Leonardi, 1951; Conti et al., 1975, 1977, 1979; Ceoloni et al., 1988; Wopfner, 1999; Avanzini et al., 2011). Plant remains were collected from several levels of this formation, including shoots, leaves, trunks and fructifications belonging to horsetails, seed ferns, cycads, ginkgophytes, conifers and some unclassified taxa (Leonardi, 1948; Klaus, 1963; Vischerr & Brugman, 1988, Pittau, 2005; Kustascher et al., 2012). Palynology suggests a late Capitanian to Changhsingian age (Pittau, 2005) or a Lopingian age (Visscher, in Kustascher et al., 2012; Posenato, 2010).

References

Avanzini M, Bernardi M, Nicosia U (2011) The Permo-Triassic tetrapod faunal diversity in the Italian Southern Alps. In: Ahmad Dar I, Ahmad Dar M, editors. Earth and Environmental Sciences. pp. 591−608.

Avanzini M, Breda A, Kustatscher E (2010) Day 1: The Permo-Triassic succession at Bletterbach Gorge. In: Gianolla P, Avanzini M, Breda A, Kustatscher E, Preto N, Roghi G, Furin S, Massari F, Picotti V, Stefani M, editors. Dolomites. 7th International Triassic Field Workshop: Pan-European Correlation of the Triassic. Field trip to the World heritage site of the tethyan Triassic. pp. 20−59.

Broglio Loriga C, Neri C, Pasini M, Posenato R (1988) Marine fossil assemblages from Upper Permian to lowermost Triassic in the western Dolomites (Italy). Mem Soc Geol It 34: 5–44.

Cassinis G, Cortesogno L, Gaggero L, Massari F, Neri C, Nicosia U, Pittau P (1999) Stratigraphy and facies of the Permian deposits between Eastern Lombardy and the Western Dolomites. Field Trip Guidebook. International Field Conference of “The Continental Permian of the Southrn Alps and Sardinia (Italy)”. Regional Reports and General Correlations”, pp. 157.

Ceoloni P, Conti MA, Mariotti N, Nicosia U (1988) New late Permian tetrapod footprints from the Southern Alps. Mem Soc Geol It 34 (1986): 45–56.

Conti MA, Leonardi G, Mariotti N, Nicosia U (1975) Tetrapod footprints, fishes and molluscs from the Middle Permian of the Dolomites (N. Italy). Mem Geopal Univ Ferrara 3: 139–150.

Conti MA, Leonardi G, Mariotti N, Nicosia U (1977) Tetrapod footprints of the “Val Gardena Sandstone” (North Italy). Their paleontological, stratigraphic and paleoenvironmental meaning. Palaeont It NS 40: 1−91.

Conti MA, Leonardi G, Mariotti N, Nicosia U (1979) Nuovo contributo alla stratigrafia delle “Arenarie di Val Gardena”. Mem Soc Geol It 20: 357−363.

Klaus W (1963) Sporen aus dem südalpinen Perm. Jahrb Geol Bund Wien 106: 229–363.

Kustatscher E, van Konijnenburg-van Cittert JHA, Bauer K, Butzmann R, Meller B, Fischer TC (2012) A new flora from the Upper Permian of Bletterbach (Dolomites, N-Italy). Rev Palaeobot Palynol 182: 1–13.

Leonardi P (1948) Contributi alla conoscenza delle flora delle Arenarie di Val Gardena (Permiano medio-inf.) dell’Alto Adige: la nuova flora di Redagno e una felce di Egna. Mem Ist Geol Min Univ Padova 16: 3–15.

Leonardi P (1951) Ricerche sulla geologia della regione dolomitica. La ricerca scientifica, 21: 783–786.

Massari F, Neri C (1997) The infill of a supradetachment(?) basin: the continental to shallow-marine Upper Permian siccession in the Dolomites and Carnia (Italy). Sed Geol 110: 181–221.

Massari F, Conti MA, Fontana D, Helmold K, Mariotti N, Neri C, Nicosia U, Ori GG, Pasini M, Pittau P (1988) The VGS and Bellerophon Formation in the Bletterbach gorge (Alto Adige, Italy): biostratigraphy and sedimentology. Mem Sc Geol 40: 229–273.

Massari F, Neri C, Pittau P, Fontana D, Stefani C (1994) Sedimentology, palynology and sequence stratigraphy of a continental to shallow-marine rift-related succession: upper Permian of the Eastern Southern Alps (Italy). Mem Soc Geol 46: 119–243.

Morelli C, Bargossi GM, Mair V, Marocchi M, Moretti A (2007) The lower Permian volcanics along the Etsch valley from Meran to Auer (Bozen). Mitt Österr Miner Ges 153: 195–218.

Neri C (2007) Arenaria di Val Gardena. In Cita MB, Abbate E, Balini M Conti MA, Falorni P,

Ori GC (1988) The nature of Permian rivers in Southern Alps. In: Cassinis G, editor. Permian and Permian-Triassic boundary in the South-alpine segment of the western Tethys and additional regional reports. Mem Soc Geol It 34: 155–160.

Pittau P (2005) The microflora. In: Pittau P, Kerp H, Kustatscher E, editors. The

Bletterbach canyon: “Let us meet across the P/T boundary” - workshop on Permian and Triassic Palaeobotany and Palynology, Bozen 16.-18.06.2005, Excursion guide. pp. 9–19.

Posenato R (2010) Marine biotic events in the Lopingian succession and latest Permian extinction in the Southern Alps (Italy). Geol Journal 45: 195–215.

Richthofen F von (1860) Geognostische Beschreibung der Umgegend von Predazzo, S. Cassian und der Seisser Alpe in Südtirol. Perthes, Gotha.

Visscher H, Brugman WA (1988) The Permian-Triassic boundary in the southern Alps: A palynological approach. Mem Soc Geol It 34: 121–128.

Wopfner H (1999) Über Tetrapoden-Fährten, Kohlen und versteinerte Hölzer aus dem Grödner Sandstein (Perm) bei Deutschnofen. Der Schlern 73: 23–32.
