# Supplementary material for: The Origin and Early Radiation of Archosauriforms: Integrating the Skeletal and Footprint Record
Source: PLoS One. 2015 Jun 17;10(6):e0128449. doi: 10.1371/journal.pone.0128449 (PMC4471049; doi:10.1371/journal.pone.0128449)
Supplement: S2 Text — (DOCX) [file pone.0128449.s004.docx]

**Supporting information**

Bernardi et al. The origin and early radiation of archosauriforms: integrating the skeletal and footprint record. PlosOne.

**S2**

Ichnostratigraphic remarks

Klein and Haubold (2007) and Klein and Lucas (2010) in an attempt to establish a series of biochronological units for the Triassic erected a “*Protochirotherium* (*Synaptichnium*) biochron” with a first appearance datum (FAD) in the Late Induan and preceding the first occurrence of footprint assemblages characterized by *Chirotherium barthii* in the Anisian. They recognized *Protochirotherium* as a characteristic morphotype with wide spatial and restricted temporal distribution and therefore an ideal index ichnofossil for the Early Triassic time interval. The skeletal record of basal archosauriforms led Klein et al. (2013) to hypothesize a possible extension of the stratigraphic range of *Protochirotherium* into the earliest Triassic or even into the Permian. The findings here support this hypothesis and extend the lower boundary of the *Protochirotherium* biochron into the Late Permian.

References

Klein H, Haubold H (2007) Archosaur footprints – potential for biochronology of Triassic continental sequences. In: Lucas SG, Spielmann JA, editors. The Global Triassic. New Mexico Mus Nat Hist Sci Bull 41: 120–130.

Klein H, Lucas SG (2010) Tetrapod footprints – their use in biostratigraphy and biochronology of the Triassic. Geol Soc London S Pub 334: 419–446.

Klein H, Niedźwiedzki G, Voigt S, Lagnaoui A, Hminna A, Saber A, Schneider JW (2013) The Tetrapod Ichnogenus Protochirotherium Fichter and Kunz 2004, a characteristic Early Triassic morphotype of central Pangea. Ichnos 20: 24–30.
