# Supplementary material for: The Origin and Early Radiation of Archosauriforms: Integrating the Skeletal and Footprint Record
Source: PLoS One. 2015 Jun 17;10(6):e0128449. doi: 10.1371/journal.pone.0128449 (PMC4471049; doi:10.1371/journal.pone.0128449)
Supplement: S3 Text — (DOCX) [file pone.0128449.s005.docx]

**Supporting information**

Bernardi et al. The origin and early radiation of archosauriforms: integrating the skeletal and footprint record. PlosOne.

**S3**

*Archosaurus rossicus* body length extimation

The skull length of *Archosaurus rossicus* was estimated using an equation derived from a linear regression of skull length versus premaxilla body height. Ten proterosuchid specimens from the *Lystrosaurus* Assemblage Zone of South Africa (*Proterosuchus fergusi*) and China (‘*Chasmatosaurus*’ *yuani*) were used for this regression (see Ezcurra & Butler, 2015; Table SV1). The regression showed a R^2^ of 0.823, a p-value<0.001, and the obtained equation was y = 0.0439x - 0.4117.

The estimated skull length of *Archosaurus* is of 460 mm and Ezcurra and Butler (2015) estimated a total body length of 3−3.5 m for the largest specimens of *Proterosuchus fergusi* (skull length=477 mm). As a result, it can be inferred that the total body length of *Archosaurus* was around 3−3.5 m.

| **Specimen** | **Skull length** | **Pmx body height** | **Pmx body length** |
| --- | --- | --- | --- |
| RC 59 | 177.6* | 6.0 | 21.0 |
| BP/1/4016 | 238.5* | 8.5 | ? |
| SAM-PK-K140 | 287.0 | 15.1 | 36.1 |
| SAM-PK-11208 | 350.0 | 17.5 | 55.9 |
| BP/1/3993 | 388.0 | 17.7 | 53.6 |
| RC 846 | 420.6 | 15.7 | 53.2 |
| BSPG 1934 VIII 514 | 435.0 | 17.1 | ? |
| IVPP V4067 | 436.0 | 20.8 | 72.6 |
| TM 201 | 443.6* | 17.0 | 51.6 |
| GHG 231 | 477 | 20.9 | 78.2 |
| Holotype of *Archosaurus* | X | 19.8 | 73.6 |

**Table S3.** Skull length, premaxilla body height and premaxilla body length of proterosuchid specimens used to estimate the total skull length of *Archosaurus*. Premaxilla body length is indicated only for comparative purposes and was not used for the estimation. Skull lengths indicated with an asterisk (*) were estimated by Ezcurra & Butler (2015). Abbreviations: ?, unavailable measure due to breackage; Pmx, premaxilla; X, value to be estimated by the linear regression.

Reference

Ezcurra MD, Butler RJ (2015) Taxonomy of the proterosuchid archosauriforms (Diapsida: Archosauromorpha) from the earliest Triassic of South Africa, and implications for the early archosauriform radiation. Palaeontology 58: 141–170.
